# Shedding Light Onto the Nature of Iron Decorated Graphene and Graphite Oxide Nanohybrids for CO_2_ Conversion at Atmospheric Pressure

**DOI:** 10.1002/open.201900368

**Published:** 2020-02-14

**Authors:** Rhodri E. Owen, Fernando Cortezon‐Tamarit, David G. Calatayud, Enid A. Evans, Samuel I. J. Mitchell, Boyang Mao, Francisco J. Palomares, John Mitchels, Pawel Plucinski, Davide Mattia, Matthew D. Jones, Sofia I. Pascu

**Affiliations:** ^1^ Department of Chemistry University of Bath Claverton Down BA2 7AY UK; ^2^ Department of Electroceramics Instituto de Cerámica y Vidrio – CSIC Kelsen 5, Campus de Cantoblanco Madrid 28049 Spain; ^3^ Department of Nanostructures and Surfaces Instituto de Ciencia de Materiales de Madrid – CSIC Sor Juana Inés de la Cruz 3, Campus de Cantoblanco Madrid 28049 Spain; ^4^ Department of Chemical Engineering University of Bath Claverton Down BA2 7AY UK

**Keywords:** graphene oxide, supramolecular interactions, nanocatalysis, CO_2_ conversion, iron oxide catalysis

## Abstract

We report on the design and testing of new graphite and graphene oxide‐based extended π‐conjugated synthetic scaffolds for applications in sustainable chemistry transformations. Nanoparticle‐functionalised carbonaceous catalysts for new Fischer Tropsch and Reverse GasWater Shift (RGWS) transformations were prepared: functional graphene oxides emerged from graphite powders via an adapted Hummer's method and subsequently impregnated with uniform‐sized nanoparticles. Then the resulting nanomaterials were imaged by TEM, SEM, EDX, AFM and characterised by IR, XPS and Raman spectroscopies prior to incorporation of Pd(II) promoters and further microscopic and spectroscopic analysis. Newly synthesised 2D and 3D layered nanostructures incorporating carbon‐supported iron oxide nanoparticulate pre‐catalysts were tested, upon hydrogen reduction *in situ,* for the conversion of CO_2_ to CO as well as for the selective formation of CH_4_ and longer chain hydrocarbons. The reduction reaction was also carried out and the catalytic species isolated and fully characterised. The catalytic activity of a graphene oxide‐supported iron oxide pre‐catalyst converted CO_2_ into hydrocarbons at different temperatures (305, 335, 370 and 405 °C), and its activity compared well with that of the analogues supported on graphite oxide, the 3‐dimensional material precursor to the graphene oxide. Investigation into the use of graphene oxide as a framework for catalysis showed that it has promising activity with respect to reverse gas water shift (RWGS) reaction of CO_2_ to CO, even at the low levels of catalyst used and under the rather mild conditions employed at atmospheric pressure. Whilst the γ‐Fe_2_O_3_ decorated graphene oxide‐based pre‐catalyst displays fairly constant activity up to 405 °C, it was found by GC‐MS analysis to be unstable with respect to decomposition at higher temperatures. The addition of palladium as a promoter increased the activity of the iron functionalised graphite oxide in the RWGS. The activity of graphene oxide supported catalysts was found to be enhanced with respect to that of iron‐functionalised graphite oxide with, or without palladium as a promoter, and comparable to that of Fe@carbon nanotube‐based systems tested under analogous conditions. These results display a significant step forward for the catalytic activity estimations for the iron functionalised and rapidly processable and scalable graphene oxide. The hereby investigated phenomena are of particular relevance for the understanding of the intimate surface morphologies and the potential role of non‐covalent interactions in the iron oxide‐graphene oxide networks, which could inform the design of nano‐materials with performance in future sustainable catalysis applications.

## Graphical abstract

**New functional two‐dimensional** (2D) and three‐dimensional (3D) nanomaterials relevant for sustainable chemistry applications have been designed and tested. This work is of relevance to enhancing our understanding of the role of supramolecular aggregation on nanotechnology and on optimised designs for future materials for energy conversion/storage as a basis for the reverse gas water shift reaction and Fischer Tropsch chemistry.

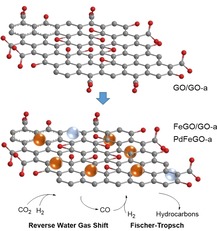

## 1 Introduction

The Climate Action Forum Paris 2015 and the recent societal concerns into the use of energy‐intensive processes highlighted that increasing environmental pressures have seen a shift in global societal attitudes and behaviours towards the use of fossil fuels for energy production, for economic reasons, as well as for communities’ livelihoods.^1^, ^2^, ^3^ It is no longer considered environmentally and socially responsible to rely solely on the burning of fossil fuels for the foreseeable future, and, as such there is a drive towards more sustainable and renewable energy solutions and their implementations in circular economies. Over the past two decades, and despite fluctuations in the commercial profile of oil‐based industries, it became an accepted paradigm that, due to the Worldwide dependence on crude oil and the depletion of other non‐renewable energy sources, coupled with the negative effects of global warming, it is necessary to find ways of producing the required energy supply in an environmentally sustainable way.^2^ This has generated interest and investment into carbon capture and storage, in a bid to reduce industrial CO_2_ emissions. CO_2_ can be viewed simultaneously as an attractive option as a feedstock towards fuels synthesis, through the conversion to methanol^4^ or via the non‐methanol mediated process using the Reverse Water Gas Shift Reaction (RWGS) and Fischer Tropsch (FT) processes. Accessing the carbon captured and stored from CO_2_ and the promise of its used towards the sustainable energy production has sparked a renewed interest in Fischer Tropsch chemistry, as this could lead to the generation of higher hydrocarbons through the use of metal catalysts and facilitate the access to this large‐scale available feedstock for a sustainable energy production.^5^

**Scheme 1:**
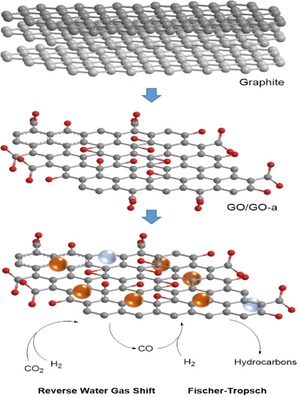
Schematic representation for the RWGS/FT processes, tested hereby on graphite and graphene oxides‐anchored Fe_2_O_3_ nanoparticles.

There are multiple alternative energy sources being investigated and there is sustained interest in novel research, which is needed to create newer ways of energy production and storage.^6^ At present no alternative energy source has the required outputs available to replace dependency on fossil fuel on a large scale.^2^ One approach would be to combine the use of processes such as solar and nuclear energy, although it has been speculated that this would require a fairly large adaptation to the current infrastructure and advancements in energy storage capabilities. However such approaches would not directly and simultaneously tackle the challenges posed by elevated CO_2_ concentration that has already been released into the atmosphere. The other approach that concerns our sustained recent research interest^7^, ^8^, ^9^, ^10^ and this study is that of the new outlook into applying nanotechnology approaches to Fischer Tropsch chemistry, additionally involving the conversion of CO_2_ to CO and the subsequent step‐wise transformation of the carbon monoxide with hydrogen into hydrocarbons.^8^ This approach has already been seen as creating a new synthetic carbon cycle – *i. e*. once hydrocarbons are burnt, the resulting carbon dioxide is then turned back into hydrocarbons.^8^, ^11^ The widely investigated FT process converts CO and hydrogen into liquid hydrocarbons. This process,^12^ typically relies on iron or cobalt ions catalysed heterogeneous catalysts.^7^ Ruthenium and nickel are also active as catalysts for hydrocarbon production; however, ruthenium is expensive and has insufficient Worldwide reserves for large scale commercial application. Under practical conditions, nickel catalysts produce mainly methane.^13^ Interestingly, CO_2_ can be used as a starting material for FT chemistry processes,^3^ albeit this has to be first hydrogenated to CO which is then utilised in the FT process (Scheme 1). Enhancing the efficiency of this process could lead to the quantity and quality of liquid hydrocarbons required in order to make the process commercially competitive with current oil prices. Due to their varying activity towards water‐gas‐shift equilibrium reactions (Eq. (A)), typically catalysed by (supported) Fe_2_O_3_ iron species are the preferred FT catalysts for transformations concerning syngas from coal plants, whereas cobalt ions as catalysts are the main choice for applications towards the conversion of natural gas derived syngas, through Eq. (B), although it is widely accepted that supported Fe_3_O_4_ heterogeneous catalysts are also highly active in the FT process (B).^14^, ^15^

(A) $$\mathrm{CO}+H_{2}O\overset{⇀}{↽}{\mathrm{CO}}_{2}+H_{2}$$

(B) $$\left(2n+1\right)H_{2}+\mathrm{nCO}\to C_{n}H_{2n+2}+{\mathrm{nH}}_{2}O$$

Metallic nanoparticles, free or anchored onto supports have been shown to be very active as catalysts due to their high ratio of surface atoms.^16^, ^17^ In addition to investigations into the role of promoters and the emergence of heterobimetallic, synergetic materials leading to the discovery of palladium‐doped iron nanoparticles with increased activity towards the water‐gas‐shift reaction,^18^ of particular interest in recent years has been the role of catalytic supports in heterogeneous nano‐catalysts design. Metal oxide frameworks and carbon based frameworks have been recently investigated for their potential in heterogeneous catalysis. Carbon based frameworks such as carbon nanotubes have been tested as supports for Fe‐nanocatalysts^8^ or Fe‐promoted heterobimetallic nanocatalysts and are favoured due to their inertness however previous studies highlighted drawbacks whereby irreducible oxides were observed when aluminium or silicon was also part of the framework.^19^, ^20^

Several allotropes of carbon such as fullerenes, carbon nanotubes (CNTs), graphene and its functionalised derivatives such as graphene oxides have garnered considerable interest over recent years.^21^ Processable and scalable graphene and graphene oxides excite the imagination of research chemists due to its many impressive properties such as high intrinsic mobility (200,000 cm^2^/Vs),^22^ high thermal (5000 Wm^−1^K^−1^), electrical conductivity,^23^ high Young's modulus (1.0 TPa)^24^ and high optical transmittance (97.7 %).^25^ Since its discovery,^24^ pristine and also functionalised graphene has become the subject of intense observations as a highly promising material with possible applications ranging from biosensors,^26^ high performance electronics,^27^ photovoltaics and composite materials.^28^ One of the key challenges in advancing the graphene‐based materials as synthetic platforms for sustainable technologies remains in the area of effective surface functionalisation and the formation of stable hybrid graphene‐based functional materials of relevance for heterogeneous catalysis, and which are amenable to surface modifications in a sustainable and straight forward manner accessible to standard chemistry laboratories. The difficulties surrounding the lab‐scale availability of functional graphenes for catalysis are associated with some considerable structural and atomic variability of the bulk‐produced materials. Popular methods of surface modification for carbon nanomaterials in general rely on covalent approaches, which, for bulk graphene require oxidised variants of graphene as a prerequisite to introducing oxygen groups and defects in the sp^2^ extended network such as epoxides, tertiary alcohols and peripheral carboxylic acids.^29^

Recent work highlighted the potential of graphene‐based frameworks as catalytic support as it has similarities to other carbon based systems such as graphitic carbon in terms of large surface area and excellent electronic conductivity, whilst also displaying some variation as the sheets have edges which have different electronic properties.^30^ Recent work has suggested that graphene oxide has a higher theoretical surface area than CNTs and due to both sides of the nano‐sheet being physically accessible, the material shows a more efficient use of surface area regarding modifications with small molecules or even nanoparticulate materials.^19^ The oxidation of graphene to graphene oxide functionalises the surface by introducing alcohol groups in the bulk and carboxylic acid groups on the edge of the structure. For tethering catalytic species onto, the benefit of these functionalities is two‐fold: the sites themselves may be catalytically active as well as providing anchoring sites for nanoparticles.

Several new examples of nano‐carbon based catalysts for the FT chemistry with activity towards the RWGS reaction has previously been generated and tested by Jones *et al*. and others, and the activity was assigned to be due to the embedded and bridging nanoparticles emerging from CVD to as‐made carbon nanomaterials or local defects in the nanotube structure.^12^, ^31^, ^32^, ^33^, ^34^, ^35^ These works highlight the ability of graphene to act as a 2D catalyst support compared to previously investigated 3‐dimensional structures such as nano‐silica and zeolites and present comparable activities with that of similar systems based on multiwalled carbon nanotubes.^10^, ^36^

## 2 Results and Discussion

A new nanohybrid displaying embedded γ‐Fe_2_O_3_ nanoparticles of consistent sizes (γ‐Fe_2_O_3_@graphene oxide) was synthesised and reduced *in situ* using H_2_ gas (at 200 °C) prior to the catalysis testing towards the CO_2_ conversion at the atmospheric pressure, as described below. A pre‐activation step using H_2_ aimed at generating Fe_3_O_4_ in situ, as these species are known to be active for both steps (A) and (B) shown above, which would render the Fe‐based nano‐hybrids as active nanocatalysts, by analogy previously‐tested and iron‐decorated carbon nanotubes.^32^ The catalytic species emerging from the *in situ* hydrogenation denoted Fe@graphene oxide and tested for the CO_2_ to CO and hydrocarbon conversion *via* FT reactions, without isolation, and using CO_2_ : H_2_ 2 : 6 ml/min feed‐stocks at 300, 335, 370 and 405 °C. In a parallel experiment the activated pre‐catalysts isolated after the hydrogenation step were collected and fully characterised.

Here we report on our advances made in the structural and morphological characterisation of the 2‐dimensional, layered nanocatalysts. For a comparison, we also used their 3D hybrid nanomaterial precursors, i. e. the graphite oxide‐based iron nanocatalysts as a control tests for the catalytic performance to investigate the potential benefits of having an oxide‐functionalised carbon framework support for the iron NPs. (Scheme 1). This approach led us to the exploitation of supramolecular chemistry‐inspired nanotechnology methods towards the development of inorganic‐organic nanoparticulate hybrid incorporating graphene‐based nanostructures.

### 2.1 Generation of 2D and 3D Nanomaterials as Synthetic Scaffolds for the Nanoparticulate Catalysts

A series of iron oxide functionalised graphene and graphite oxide pre‐catalysts were synthesised via a rapid and facile γ‐Fe_2_O_3_ nanoparticle incorporation into the functional carbon nano‐sheets, as discussed in the Experimental Section. The graphene oxide synthetic scaffold was first synthesised by the exfoliation of graphite. Subsequently iron nanoparticles (γ‐Fe_2_O_3_, synthesised as described in ESI) with well understood morphologies and dimensions (i. e. averaging 50 nm in aqueous media, as shown by dynamic light scattering DLS, shown in ESI), were introduced into the nanostructure thus yielding a novel pre‐catalyst scaffold, ready for a subsequent pre‐activation by H_2_ and CO_2_/H_2_ reactions for a rapid CO_2_ conversion.

To bypass possible drawbacks from batch to batch reproducibility, several different batches of graphene and graphite oxides were prepared as precursors and were functionalised with tailor‐made and fully characterised Fe_2_O_3_ nanoparticles (NPs), showing consistency in morphology and NPs distributions on surfaces, prior to the pre‐activation under H_2_ and catalysis testing. Specifically, graphite oxide was synthesised using an adapted Hummer's method,^37^ adapted with the use of 2 mL of 30 % H_2_O_2_. The resulting slurry was centrifuged at 3000 rpm and the resulting solid was washed at least 10 times and freeze‐dried. The procedure was repeated at least three times generating different batches of graphite oxide with high batch‐to‐batch consistency as evidenced by TEM and spectroscopic data. Both graphene oxide and graphite oxide emerging were functionalised with iron oxide nanoparticles by combining 20 %wt. of iron nanoparticles with the solid sample of carbonaceous materials in toluene followed by further re‐dispersions and drying protocols followed by full structural characterisation (ESI). Furthermore, palladium acetate (3 %wt.) and the iron oxide functionalised graphite oxide hybrid were combined in toluene and heated gently, the excess solvent was removed, the sample was washed with THF and excess solvent was removed under vacuum. The resulting pre‐catalysts were followed by *in situ* generation of the iron active species through mild hydrogenations and then their testing as nano‐catalyst for the CO_2_ activation following existing protocols. In our hands, a surprisingly short exposure time of only 40 minutes was found to give a comparable conversion rate with silica supported catalysts, and this intriguing result was assigned to the layered morphology and the accessible encapsulated iron nanoparticles within the graphene supports, *vide infra*.^10^, ^36^

### 2.2 Structural characterisation of pre‐catalysts on the nanoscale

**Figure 1:**
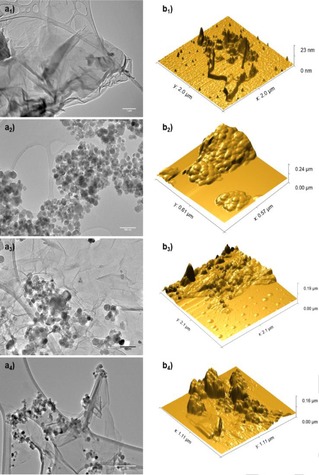
TEM (a) and AFM (b) images of GO‐a and pre‐catalyst species : (a_1_)–(b_1_) graphite oxide; (a_2_)–(b_2_) Fe_2_O_3_ nanoparticles;(a_3_)–(b_3_) Fe_2_O_3_@GO‐a and (a_4_)–(b_4_) Pd@Fe_2_O_3_@GO‐a. Scalebars: a_1_) 1 um, a_2_) a_3_) 100 nm, a_4_) 200 nm. Corresponding H_2_‐reduced species (support precursors and catalysts) have been isolated and characterised: data is given in ESI.

All nano‐particulate pre‐catalytic species were imaged on the nanoscale by tapping mode AFM, showing that the nano‐hybrid character is maintained throughout and morphologies are on the nanoscale and consistent with the observations by TEM and SEM. XRD analyses of the Fe_2_O_3_ nanoparticles and Fe‐decorated nanocarbon pre‐catalyst confirm the presence of pure crystalline γ‐Fe_2_O_3_ nanoparticles (see ESI). Interestingly, the γ‐Fe_2_O_3_ NPs were clustered on the graphitic network of **GO‐a** as well as **GO** supports, whilst maintaining their NP character in terms of shapes and size (Figure 1 and ESI).

### 2.3 Graphite Oxide Frameworks

**Figure 2:**
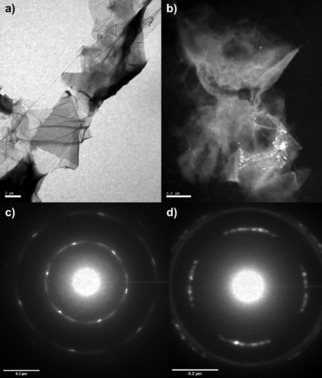
a) Graphene oxide (GO) TEM, b) GO TEM (dark field), c) Graphite Oxide (GO‐a) Electron Diffraction Pattern, d) Graphene Oxide (GO) Electron Diffraction Pattern.

**Figure 3:**
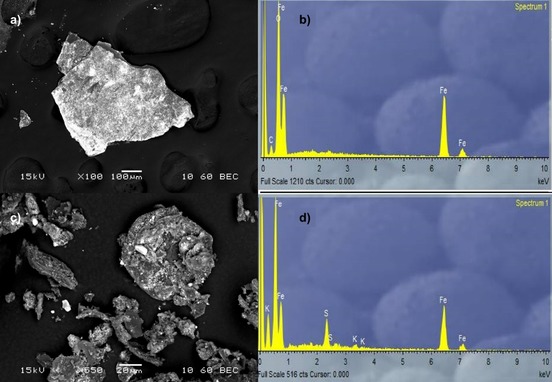
(a) The SEM of Fe_2_O_3_‐decorated graphene oxide FeGO, (b) EDX of FeGO‐a, (c) SEM of FeGO‐a showing surface morphology of this pre‐catalyst, (d) The EDX of Fe_2_O_3_‐decorated graphene oxide (FeGO).

Graphite Oxide framework (**GO‐a**), prepared and isolated as described above and in the Experimental Section, was characterised by spectroscopic methods including Raman, XPS and IR spectroscopies and imaged by TEM coupled with SAED and EDX (Figures 2 and 3). Although the **GO‐a** electron diffraction has some out of plane reflections, arranged in the hexagonal pattern that are clearly visible, leading to the conclusion that this is un‐exfoliated graphite.

The TEM images showed a darker region, assignable to the agglomeration of carbon layers, which is indicative of thick carbon layers expected from this 3D layered functional material.

The iron oxide‐functionalised graphite oxide pre‐catalyst (denoted γ‐Fe_2_O_3_@graphite oxide or **FeGO‐a**) synthesised as a new composite from the treatment of graphite oxide with γ‐Fe_2_O_3_ nanoparticles as described in the Experimental Section, was characterised by spectroscopic methods including Raman, XPS and FTIR spectroscopies in the solid state. The resulting morphology on the nanoscale was imaged by TEM (Figures 1–3). The electron diffraction pattern shows the characteristic hexagonal form of graphite, however the multitude of rings show the difference upon Fe_2_O_3_ encrustation and whilst there is also a tetragonal crystalline species present likely Fe_2_O_3_ in bulk rather than only the γ‐Fe_2_O_3_ in the nano‐particulate form. The SEM image shows that iron centres have covered some of the surface, however these areas do not appear to be distributed in an even manner. These features were consistent across the several different batches of graphene oxide (GO) synthesised from the exfoliation of graphite oxide precursors described above and used as the synthetic scaffold for heterogeneous Fe‐based nanocatalyst support. Images are also consistent with those expected for the few‐layers of GO (isolated as described in the Experimental Section) as evident from imaging by TEM and electron diffraction (ESI and Figures 1–2). The diffraction pattern of all GO batches used is indicative of a 2D material, exfoliated layers of graphene which have agglomerated together, but are placed in slightly different diffraction planes.

The TEM image suggested a very small depth and shows clearly a very thin several layer sheet of graphene. Energy dispersive X‐ray diffraction gave information on the local elemental composition this isolated material seems to show a clean sample containing mainly C, H and O elements, with minimum N‐based defects being present. The elemental analysis by EDX showed no significant impurities in either GO or its corresponding iron oxide pre‐catalysts, in addition to those already present in the graphite oxide support which suggests that an iron oxide is the most likely iron‐containing functionality present on the surface of the desired γ‐Fe_2_O_3_@graphene oxide (denoted **FeGO**).

**Figure 4:**
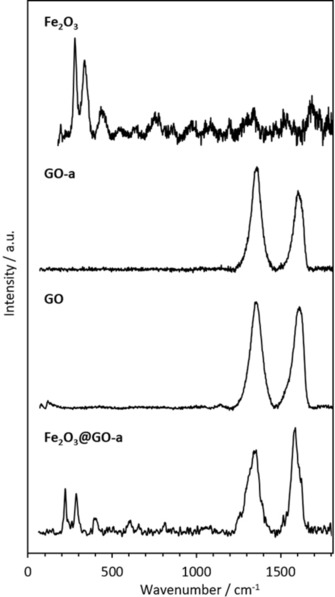
Raman spectroscopy of: Fe_2_O_3_, GO‐a, GO and FeGO‐a.

The Raman spectroscopy showed the pattern assignable to GO in solution and when deposited on a metal surface (ESI and Figure 4). Both of these samples displayed D & G bands which are characteristic of graphene 2‐D networks at 1300 & 1600 cm^−1^. These Raman bands indicate that the functional graphene type material in question is thin in layer count as the peaks are fairly sharp and especially intense when concentrated, as shown by Raman mapping using the 830 nm excitation.

**Figure 5:**
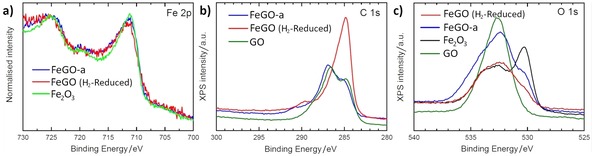
Representative high‐resolution XPS spectra corresponding to the Fe2p (a), C1s (b) and O1s (c) core levels for Fe_2_O_3_, GO‐a and FeGO‐a pre‐catalysts and FeGO‐a (H_2_‐reduced). Corresponding images for the activated catalysts are given in ESI.

XPS spectroscopy was performed to determine the surface chemical composition of the pre‐catalysts as a consequence of the interactions between the iron oxide and functionalise graphene sheets (Figure and ESI). Figure 5 shows the XPS data recorded for **FeGO‐a** with a focus on the Fe2p, C1s and O1s core levels. Figure 5a first shows the XPS spectra corresponding to the Fe2p region for the Fe_2_O_3_ and **FeGO‐a** and, as observed, both the peak, located at around 710 eV, and energy shift of the satellite between the doublet are characteristic features corresponding to the Fe_2_O_3_ species, as expected. In addition, the **FeGO‐a** spectrum shows a shoulder at higher energy side of the main peak which corresponds to emission from FeO, suggesting that the Fe_2_O_3_ particles are conjugated to the **GO‐a** surface through CO groups. Sharp peaks located at around 284.9 eV and 285.7 eV correspond to C=C/C−C in aromatic rings and C−O in hydroxyl and epoxy groups, respectively. The peak located at a binding energy of ca 290 eV corresponds to the C−O−Fe functional groups. Figure 5c show the O1s spectra for **GO‐a** and **FeGO‐a**, where two oxygen signals situated around 534 and 532 eV were detected, and be assigned to C−O−H and C−O, respectively. The incorporation of Pd into the materials does not produce any detectable change in the XPS spectra (ESI). In this sense, as the concentration of Pd in the samples is around 1 %wt, it is not possible to be detected by XPS (below the detection limit of the equipment). Sulphur is also detected over the surface of all the **GO‐a** samples, as previously observed for sample originating from Hummer's treatments.^38^

For the **FeGO** and **FeGO‐a** pre‐catalyst, detailed microscopy studies were carried out in order to observe the morphology of the starting materials and for a comparison with the desired adducts with iron‐based nanoparticles. The SEM image (Figure 3) shows that the iron nano‐particulates are distributed homogeneously across the surface of the graphene. A direct comparison of images obtained from TEM was informative for the nature of the catalyst: for graphite oxide showed areas of partially exfoliated and un‐exfoliated layers. This was to be expected as only mild sonication was carried out in the synthesis. The diffraction pattern showed the presence of large, bright spots in a hexagonal lattice, with sharp rings between spots. The clear orientation of the spots was due to the thickness of the graphite layers (>200 nm) which result in smaller diffraction. The sharp rings indicate an un‐exfoliated sample with carbon atoms all in the same plane. Comparatively, the TEM of graphene oxide showed smaller, sparser bright spots with blurred rings. This indicates that the layers have been successfully exfoliated and are rotating in opposite directions, giving a greater overall diffraction. TEM shows that hexagonal geometry is maintained consistent with the formation of a 2D carbon graphene‐type nanostructure. The electron diffraction pattern was used to measure the d spacing, graphite oxide d=2.01 Å, graphene oxide d=2.91 Å, and Fe‐GO nanomaterial synthesised hereby had a spacing of 1.88 Å as indicated by electron diffraction.

There are clear structural differences observed for the GO‐a, GO and corresponding Fe oxide‐decorated 3D vs 2D nano‐hybrid species: as expected, selected area electron diffraction (SAED) of free graphene oxide support (GO) indicates a crystalline structure while its direct observation by TEM reveals a sheet‐like nano‐material. TEM micrographs of the Fe‐oxide decorated graphene oxide shows a heterogeneous distribution of the nanoparticles with various degrees of aggregation, consistent with the AFM investigations (Figure 1). In all batches analysed, imaging showed well dispersed sheets of the graphene oxide nanohybrid complex, while in a different site of the sample it can be observed a multilayer aggregation structures. EDX analysis of all batches of supports investigated and nano‐hybrid pre‐catalysts samples confirmed the presence of the iron together with carbon species and, therefore, indicated the formation of the desired nano‐complexes (ESI). XPS spectroscopy show no significant differences were observed for the **GO** and **FeGO** samples, which have similar XPS spectra than **GO‐a** and **FeGO‐a**.

The understanding of the morphology of the Fe‐based graphite oxide was crucial, as this was chosen as a benchmark complex which was chosen to be tested in the RWGS reaction (*vide infra*) was also investigated by TEM. In this case the TEM indicates the formation of crystalline presumably iron‐based nanoparticles of the relevant hybrid together with amorphous material. The presence of the iron, together with carbon species was confirmed by EDX analysis (see ESI). The morphology of the pre‐catalysts was also investigated on the micro‐scale by SEM together with EDX (Figures 2 and 3 and ESI).

The SEM micrograph of all iron‐decorated systems shows a sheet‐like morphology while EDX analysis confirm the presence of both Fe and GO‐specific functionalities (O, N, C) (ESI). These results indicate that both graphene oxide and its precursor graphite oxide can be functionalised with Fe‐containing nanoparticles. Interestingly, single layers of functionalised carbon nanosheets can be observed for the graphene oxide‐based adducts. However, a certain degree of aggregation and a presence of amorphous carbonaceous materials can be seen for all samples, across all batches, which would make the assignment of functionalities present and level of oxygenation hard to estimate, but which would likely have an effect on the level at which the CO_2_/H_2_ feedstock and the emerging products including CO are absorbed onto the pre‐catalysts. The electron diffraction of the iron modified carbon nanostructure used as a nanocatalyst precursor showed bright spots and clear circular rings indicative of unexfoliated carbon layers for graphite oxides/graphene oxides as expected. The image also showed the presence of a cubic lattice corresponding to Fe_2_O_3_ deposited on the carbon layers. TEM shows an un‐exfoliated graphite flake with good coverage of iron nanoparticles, ranging in size from 20–40 nm.

**Figure 6:**
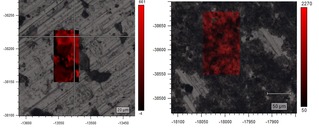
Raman mapping of intensity at 1440 cm^−1^ for GO and FeGO pre‐catalysts.

The differing brightness of the nanoflake imaged indicated unexfoliated local areas (dark) and partially exfoliated areas (lighter). SEM imaging showed Fe nanoparticles (bright spots) on graphite oxide (dark region). Interestingly, the electron diffraction of un‐modified graphene oxide showed the blurred rings indicated exfoliated layers rotating in opposite directions. Interestingly, the Raman spectroscopy performed indicated that the iron oxide was distributed over the surface, but not on the edges of the graphene sheets, as bands characteristic for the iron oxide were not observable with the laser focused on the edge, however the presence of iron oxide was apparent when a wider focus on the bulk nanomaterials sheets was used. The increase in ratio of D to G peak is in line with all functionalised graphenes (Figure 4). The Raman mapping (Figure 6) showing the peak intensities for 1440 cm^−1^ endorses the observations on the nanoscale and are consistent with the AFM imaging (Figure 1).

Insights into the morphology and nature of the reduced species post hydrogenation treatment were obtained through combined AFM, SEM andTEM techniques. Surface studies do not show dramatic differences between starting materials and the corresponding pre‐catalyst samples, post hydrogen‐treatment however this is not surprising as the hydrogen activation involved rather mild conditions. Morphology of the activated species, when compared to the pre‐catalysts above studied by TEM and AFM are similar, however, as expected, reduced GO samples appeared somewhat more aggregated upon inspection by AFM but this is sample and region dependent. A qualitative comparison of the results from EDX maps did not show significant differences in terms of the level of oxygenation, as expected, and these are dominated by contribution from the carbon support and copper from the grids, whilst the comparison of such heterogeneous areas in terms of their relative oxygenation is not reliable.

Intriguingly, the metallic nanoparticles on the surface of the reduced [PdFeGO‐a] sample seem to present a coating, observed by TEM, yet not previously present in the starting material PdFeGO‐a or in the other hydrogen‐reduced samples such as [FeGO‐a] or [FeGO]. The resolution of EDX was insufficient to reveal local composition in detail, however previous studies indicated that Lewis acid promoters facilitate formation of iron carbide as the active species.^7^ This hypothesis was not validated hereby and Raman spectroscopy of these species did not support these assignments.

Specific surface are measurements conducted for the pre‐catalysts as well as the corresponding H_2_‐reduced species were carried out by nitrogen adsorption/desorption isotherms.

Table 1 shows that the incorporation of the Fe_2_O_3_ over GO‐a and GO supports produces an increase in the specific surface area of the material. The sample FeGO‐a shows the highest specific surface area, which can be justified due to GO‐a preserves the layer structure of the parent graphite, with interlayer porosity. By contrast GO is a 2D material without interlayer porosity. These values can provide an idea about the density of active sites. Although FeGO‐a and PdFeGO‐a show the highest values specific surface area (Table 1 and ESI), part of the specific surface area is due to the interplanar porosity, which is not accessible for the Fe_2_O_3_ nanoparticles, suggesting that the samples denoted FeGO‐a, PdFeGO‐a and PdFeGo have very similar population of substrate‐accessible catalytic active sites.

**Table 1:** Specific surface area of the prepared samples and corresponding pore size.

Fe‐decorated Nanocarbon Pre‐catalyst	Surface Area (m^2^/g)
Fe_2_O_3_	21
GO	197
GO‐a	211
FeGO	221
FeGO‐a	234
PdFeGO‐a	234
[FeGO] (H_2_‐Reduced)	222
[FeGO‐a] (H_2_‐Reduced)	224
[PdFeGO‐a] (H_2_‐Reduced)	225

### 2.4 *In situ* Hydrogenation of Nanohybrid Pre‐Catalysts and RGWS/FT Reactions Testing

Given that the chemical modification of the surface of graphene layers leads to its decoration with oxygenated functional groups and introduction of defects into the planar graphitic layers (as evidenced by XPS and Raman spectroscopies), this in turn allows the anchoring of Fe_2_O_3_ nanoparticles onto the surface of the graphitic layers, through covalent C−O−Fe interaction, hydrogen bonding and nano‐encapsulation due to aromatic stacking of the GO, as also evidenced by XPS (ESI).

Spectroscopic investigations outlined above allowed a comparison between the pre‐catalyst morphologies and the starting materials, *i. e*. pristine graphene oxide and graphite oxide, *vide infra*. The active nano‐catalytic species for both were generated via an *in situ* hydrogenation procedure of the FeGO‐a and FeGO pre‐catalysis, and the resulting H_2_‐activated species denoted reduced[FeGO‐a] and reduced [FeGO] were used in the catalysis tests for the reactions (A) and (B) above. This approach was already developed as a rapid and efficient route to the *in situ* generated active nanocatalysts in the case of iron or cobalt pre‐catalysts supported on a variety of inorganic scaffolds and pristine or N‐doped multi‐walled carbon nanotubes.^31^ The activity of graphene oxide modified catalysts could be compared to that of iron modified graphite oxide: experiments suggest that graphene oxides are a promising support in order to generate a more active and efficient catalyst than its graphite counterparts for the reduction of CO_2_ to CO and subsequently of CO to hydrocarbons. The products yielded using the iron modified graphite oxide catalyst indicated mainly the formation of CO.

At the low temperatures tested hereby (300, 335, 370 °C) all catalysts showed good conversion and selectivity (Table 2). However at 405 °C, the graphitic structures began to decompose to give aromatic products, benzene, α‐methyl styrene, and ethyl benzene, as seen from the GC‐MS (FID detector).

**Table 2:** Catalysis data obtained for CO_2_ hydrogenation tests using the pre‐catalysts Fe_2_O_3_‐supported on graphite oxide or graphene oxide, over a range of temperatures (residence time 40 min).

Fe‐decorated Nanocarbon Pre‐catalysts	T (°C)	Total CO_2_ Conversion (%)	Hydrocarbon Selectivity (%)					
			Methane (1)	Ethene (2)	Ethane (3)	Propene (4)	Propane (5)	C4+ (6)
Fe_2_O_3_@Graphene Oxide (FeGO)	300	6.7	100	–	–	–	–	–
	335	11.9	81.7	5.6	3.6	3	2.5	3.4
	370	12.7	86.7	4.3	3	2.2	1.9	1.9
	405	11.7	86.9	4.7	4.2	2.7	1.5	–
Fe_2_O_3_@Graphite Oxide (FeGO‐a)	300	9.1	67.1	4.4	7.2	8.5	5	7.9
	335	14.4	81.2	7.6	4.8	4	2.4	–
	370	15.3	65.2	9	8.1	11.4	6.3	–
	405	9.8	82	10.4	7.6	–	–	–
Pd(II)/Fe_2_O_3_@Graphite Oxide (PdFeGO‐a)	300	16.4	–	–	–	–	–	–
	335	15.1	100	–	–	–	–	–
	370	21.4	49.7	12.8	13	8.6	8	7.9
	405	20.2	62.2	8.5	9.7	5.8	6.6	7.2

A comparison of conversion and hydrocarbon selectivity for Fe‐based Graphite (FeGO‐a), Pd‐promoted Fe‐based graphite (PdFeGO‐a) and Fe‐Graphene (FeGO) catalysts at different temperatures and atmospheric pressure is shown in Table 2. Data showed that conversion of CO_2_ in total led to mainly CO formation, and conversion of CO_2_ to hydrocarbons was rather low, typically in 1 % overall, with the exception of the case of [PdFeGO‐a] nanocatalyst (formed in situ after the hydrogenation of PdFeGO‐a pre‐catalyst), where the total conversion at 300 °C was due to RWGS transformation to CO only (16 %), unlike with the graphite oxide catalyst, the graphene support showed enhanced stability at higher temperatures (405 °C) as no aromatic compounds were formed as observed by GC‐MS. Also, no alcohols or oxygenated products have been observed at these rather lower temperatures tested, but extensive degradation of the graphene oxide support was observed above 405 °C. The most promising catalyst for this transformation was the Fe supported on graphite oxide with Pd as an activator, which is not surprising as Pd(II) is a known hydroformylation and hydrogenation catalyst.

In order to probe the reduction effects of H_2_ upon FeGO, FeGO‐a and PdFeGO‐a pre‐catalysis, XPS measurements were carried out on the isolated reduced pre‐catalyst species. Although some investigations into the use of graphenes in FT chemistry have been reported for related transformations, the synergetic role of Fe_2_O_3_/GO and their simultaneous reduction in heterogeneous catalysis has not been elucidated thus far. Several earlier publications reported the use and characterisation of reduced graphene oxide and partially reduced graphene oxide as catalyst support in a number of different catalytic processes^39^, ^40^, ^41^, ^42^ and here we used XPS for the characterisation of the degree of reduction of GO under similar conditions but applied to RGWS/FT processes. Whilst the C and O functionalities of the support were affected hereby the Fe and Pd valence states of the catalysts available on the surface were less conclusive. These results are comparable with those of the Fe‐based FTS catalysts supported on reduced graphene oxide however the conditions are significantly milder and the oxygenated groups present on the surface nearly removed: therefore the reduced graphene oxide showed selectivity for C8+ as previously demonstrated.^40^

Results indicate that the reduction under the relatively mild conditions employed (a flow of H_2_ at 50 sccm at 300 °C for 2 hours) seem only affects to the surface functionalities of GO, and not lead to the reduction of Fe_2_O_3_ particles (see Figure 5 and ESI) on the surface. Lack of observable Fe_3_O_4_ or Fe_2_C in these samples by XPS investigations may also be assignable to the presence of the oxygenated functionalities present in excess on the surface of the graphene/graphite oxides support and further studies are necessary to identify the optimum conditions to reduce both the graphene oxides and the Fe_2_O_3_ in situ.

Nevertheless, the graphene based catalyst (FeGO) was highly active in converting CO_2_ feedstock to CO even at these rather low temperatures investigated (as per eq. A);. Table 2 shows that the CO_2_ total conversion of FeGO is lower than **FeGO‐a** both at 300 °C (6.7 % vs 9.1 %) and 335 °C (11.9 % vs 14.4 %). From GC‐MS there were indications that the graphite derivative denoted FeGO‐a decomposed at higher temperatures with formation of benzene among other unexpected peaks appearing. This was not seen with the PdFeGO‐a, which could suggest added stability, GC‐MS analysis also showed that Fe doped graphite oxide had a higher conversion of CO_2_ to CO and hydrocarbons and had a higher selectivity for long chain hydrocarbons at temperatures 300–370 °C, with both catalysts showing greatest conversion at 370 °C (Table 2). At 405 °C the graphene oxide‐based catalyst FeGO achieved a higher conversion than its graphite analogue, of 11.7 % compared to 9.8 %. The catalyst also maintained a higher selectivity to long chain hydrocarbons, still producing C1−C4 whereas graphite oxide‐based counterpart was only selective to C1−C2 species. The GC‐MS analysis indicated the presence of benzene, α‐styrene and ethyl benzene for FeGO‐a and PdFeGO‐a at the higher temperature tested (405 °C). The presence of these compounds is likely due to the graphite framework breaking down at higher temperatures to yield various aromatic products. This indicated the superior stability of functionalised graphene oxide in FeGO as the support, as this was still producing a small amount of hydrocarbons at 405 °C, as well as its higher activity as a Fe‐based RGWS catalyst. We assign this to the ability of the GO layered system to encapsulate / bind to the iron oxide nanoparticles and thus achieve higher concentration of nanoparticles both in bulk and on the surface. The fact that the results were comparable across the catalysts used shows the superiority of graphene as a support structure for catalysis, more than likely due to its large surface area.^15^

Our catalysis tests also showed that further modification of the graphite oxide pre‐catalysts with palladium enhanced the catalytic activity of FeGO‐a, as expected,^7^ and its role of a promoter has been demonstrated in earlier studies. This Pd(II) promoted catalyst was most active in the series albeit at the higher temperatures (>370 °C) where it gave the higher conversions and hydrocarbon selectivity. Table 2 shows that the total conversion of CO_2_ was high for all catalysts (albeit leading mainly to CO) and compared well with the similar systems such as those encountered for carbon nanotubes (ESI).

This implies that the catalyst was most active for the first step of the reaction, the reverse water gas shift process (A), but only moderately active for the second step of the process (B) at these reduced temperature, possibly due to persistence of the Fe_2_O_3_ species after the hydrogen activation, or the degradation of the graphite oxide 3D structure, or the ineffective formation of Fe_3_O_4_ species due to the oxygenated functionalities of the graphite/graphene oxides used.

Taken together with the catalysis tests, these structural and morphological testing suggest that the particular features of the resulting Fe‐graphene oxide composite have the potential to act in a synergistic way, and operate in the rather less extreme regime compared to traditional carbon or silica‐supported FT catalysts of particular relevance as RGWS nano‐catalysts. The majority of heterogeneous nano‐catalysis investigations reported to date involving carbon nanomaterials have focused on the use of multi‐walled carbon nanotubes as supports: these results are unique in the field due to combination of nanoparticulate Fe_2_O_3_ deposited onto 2D and 3D nano‐carbonaceous supports and also comparable in terms of the activity of graphene oxide with that of CNTs, as evidenced by earlier tests under analogous conditions. Work is underway in our laboratories we are now focused on scale‐up investigations as well as the further modification of graphene oxide using a number of different Lewis acids as promoters as well as new catalytic metals, such as doping of graphene oxides with cobalt and molybdenum nanoparticles of controlled dimensions. For the nano‐carbon materials to be successfully implemented as a promising nano‐catalytic supports, the major hurdle remains in the development of a large scale synthetic routes for processable graphenes. This is made particularly difficult due to the high batch sensitivity of most synthetic carbon‐based nanoparticles such as carbon nanotubes. As such, work on large scale synthesis using simpler, more versatile supports such as graphene oxide is fundamental in paving the way to a successful future application of graphene or carbon nanotubes in hi‐tech industries and we addressed synthetic challenges hereby.

## 3 Conclusions

Hereby we report on our recent explorations regarding the structural features which render the graphene oxide as a promising framework for nanoparticulate and supported FT heterogeneous chemistry. Some novel iron‐oxide decorated nano pre‐catalysts have been synthesised hereby and their catalytic activities compared for the two step conversion of carbon dioxide and hydrogen into hydrocarbons. Nanotechnology approaches to sustainable catalysis are challenging due to the high batch sensitivity of carbon‐based nanomaterials such as carbon nanotubes, but it was mitigated here by the used of graphene oxide starting materials emerging from established and scalable synthetic protocols. Our microscopic imaging results across several different scales, from molecular‐level investigations by XRD, to nanometre scale imaging by AFM, TEM and SEM showed, on several different batches, that the particulate 3D vs 2D morphology is consistent and is maintained upon functionalisation with Fe_2_O_3_ nanoparticles of well‐understood shapes and dimensions. Work on large scale synthesis is fundamental to the successful implication of graphene and graphene oxide in hi‐tech industries. The graphene oxide modified with Fe_2_O_3_ nanoparticles showed the highest activity when considering mass of catalyst used. The majority of work on carbon nanostructures to date has focused on carbon nanotubes as a catalyst support, and we found that the activity of graphene oxide over only 40 minutes of testing under atmospheric pressure is comparable with that reported by us earlier for Fe@CNTs. These approaches could become a useful tool in the fabrication of catalytically active inorganic nanomaterials, as well as those carbon‐based and graphene‐like material employed nowadays.

## Experimental Section

All starting materials were purchased from Sigma‐Aldrich and used without further purification. All solvents were of reagent grade quality,purchased from Sigma‐Aldrich and used as received.

**Synthesis of Graphite Oxide (GO‐a)**: Graphite powder (2.0 g) and sodium nitrate (1.0 g) were added to 46 mL of concentrated sulphuric acid. The mixture was kept below 20 ^o^C and potassium permanganate was added slowly (6.0 g). The mixture was then stirred at room temperature for 30 minutes, then 90 mL of distilled water water were added slowly. After diluting the solution to 300 mL with distilled water, 2 mL of 30 % (v/v) hydrogen peroxide was added whilst stirring. The mixture was allowed to settle and the aqueous layer decanted. The solid graphite oxide was then washed, via re‐dispersing in de‐ionised water and centrifuged at 3500 rpm for 30 minutes, the resulting supernatant was decanted. The washing procedure is repeated a minimum of 10 times, the final solid was then dried in a freeze dryer.

**Synthesis of Graphene Oxide (GO)**: Graphite oxide was dispersed in 100 mL of 2 : 1 distilled water : ethanol and sonicated 6 times for 30 minutes each, allowing for the solution to stand for 10 minutes between cycles. The mix was then centrifuged at 3500 rpm for 30 minutes, removing graphite oxide from solution, leaving a suspension of graphene oxide.

**General method for the incorporation of Iron nanoparticles** within the carbon nanomaterial supports Graphite (FeGO‐a) and Graphene Oxide (FeGO): The solid support synthesised as above was dispersed in toluene and mixed with solid gamma‐Fe_2_O_3_ nanoparticles (from Aldrich, 20 %wt of Fe_2_O_3_ with respect to the carbon material used). This mixture was then sonicated for 30 minutes and left stirring for 48 hours. The resultant solution was then gently heated whilst stirring to remove the toluene initially added. The remaining black slurry was then heated to 270 °C for one hour and allowed to dry under air.

**Hetero‐bimetallic Palladium/Iron‐Modified Graphite Oxide (PdFeGO‐a)**: Palladium acetate (0.0063 g) was dissolved in toluene with the iron modified graphite oxide catalyst FeGO‐a synthesised as above (0.21 g). The mixture was then heated gently whilst stirring to remove the toluene, dried under reduced pressure and analysed by AFM, TEM and FTIR.

**Samples preparations for analysis**: For TEM analysis, samples were dispersed in methanol or ethanol and sonicated mildly (less than 5 min) to ensure dispersion. The samples were then placed on a carbon film coated copper grid. TEM was performed on a JEOL JEM 1200 EXII operated at 120 kV. For Raman spectroscopy analysis: samples were dispersed in either ethanol or methanol and a background spectrum was run against pure solvent. Samples were analysed on a Renishaw InVia Raman Microscope. For SEM coupled with EDX and XPS analysis: Samples were attached to metal stubs using adhesive carbon tape. Samples were analysed using a JEOL JSM 6480 LV Scanning Electron Microscope and SPECS Phoibos150 XPS spectrometer equipped with 2D‐DLD detector. The specific surface area was determined by the Brunauer‐Emmett‐Telle (BET) method on an ASAP 2020‐Micromeritics (Norcross, GA, USA) at 77 K . Samples were degassed at 30 °C during 48 h before analysis

**Catalysis testing**: The catalysis rig used consisted of a Carbolite furnace 90 cm in length with an internal diameter of 4 cm. Catalyst bed length was a 10 cm Swagelok 1/4 inch piping and gas flow controlled by digital omega mass flow controllers. Gas flow was controlled via Omega mass flow controllers. For GCMS analysis, gas samples were collected from catalysis rig using a gas syringe. Samples were analysed by Agilent Technologies, 7890A GC which was calibrated using the procedure detailed elsewhere.^5^

**Catalyst Testing was performed according to** the experimental setup published earlier^5^ Samples were ground up and mixed with 2 g of silicon carbide (SiC). Samples were packed into 1/4 inch (internal diameter) Swagelok stainless steel tubing with quartz wool on either end of the sample. The catalysts were subsequently loaded into a catalytic fixed bed reactor. Before the catalytic testing against CO_2_ activation, it was necessary to reduce the catalyst under a pure flow of H_2_ at 50 sccm at 300 °C for 2 hours. Each activated nano‐catalyst batch was then exposed in situ to a flow of CO_2_ (2 sccm) and H_2_ (6 sccm) at four temperatures: 300 °C, 335 °C, 370 °C and 405 °C for 40 minutes. The product mixtures were then analysed using gas chromatography mass spectrometry (GCMS). In a parallel experiment, after the H_2_‐activation process as described above the catalyst samples were isolated and characterised by AFM, TEM/EDX and XPS, and results were compared to those of the as‐made pre‐catalysts.

## Supporting information

As a service to our authors and readers, this journal provides supporting information supplied by the authors. Such materials are peer reviewed and may be re‐organized for online delivery, but are not copy‐edited or typeset. Technical support issues arising from supporting information (other than missing files) should be addressed to the authors.

Supplementary
