# Supplementary material for: Shedding Light Onto the Nature of Iron Decorated Graphene and Graphite Oxide Nanohybrids for CO2 Conversion at Atmospheric Pressure
Source: ChemistryOpen. 2020 Feb 14;9(2):242–52. doi: 10.1002/open.201900368 (PMC7020623; doi:10.1002/open.201900368)
Supplement: Supplementary file 1 — Supplementary [file OPEN-9-242-s001.pdf]

Open Access
ChemistryOPEN

Supporting Information

Shedding Light Onto the Nature of Iron Decorated Graphene and Graphite Oxide Nanohybrids for CO_2 Conversion at Atmospheric Pressure

Rhodri E. Owen, Fernando Cortezon-Tamarit, David G. Calatayud, Enid A. Evans, Samuel I. J. Mitchell, Boyang Mao, Francisco J. Palomares, John Mitchels, Pawel Plucinski, Davide Mattia, Matthew D. Jones,* and Sofia I. Pascu*© 2020 The Authors. Published by Wiley-VCH Verlag GmbH & Co. KGaA. This is an open access article under the terms of the Creative Commons Attribution License, which permits use, distribution and reproduction in any medium, provided the original work is properly cited.This article is part of our Special Collection dedicated to Functional Supramolecular Systems

Author Contributions

D.G.-C. Formal analysis:Equal; Investigation:Equal; Methodology:Equal
E.E. Data curation:Equal; Formal analysis:Equal; Investigation:Equal; Methodology:Equal; Validation:Equal
S.M. Data curation:Equal; Formal analysis:Equal; Methodology:Equal; Validation:Equal
F.P. Formal analysis:Equal; Investigation:Equal; Methodology:Equal
J.M. Data curation:Equal; Formal analysis:Equal; Investigation:Equal; Methodology:Equal

Figure S1. As-made Fe_2O_3 nanoparticles characterisation by HRTEM.

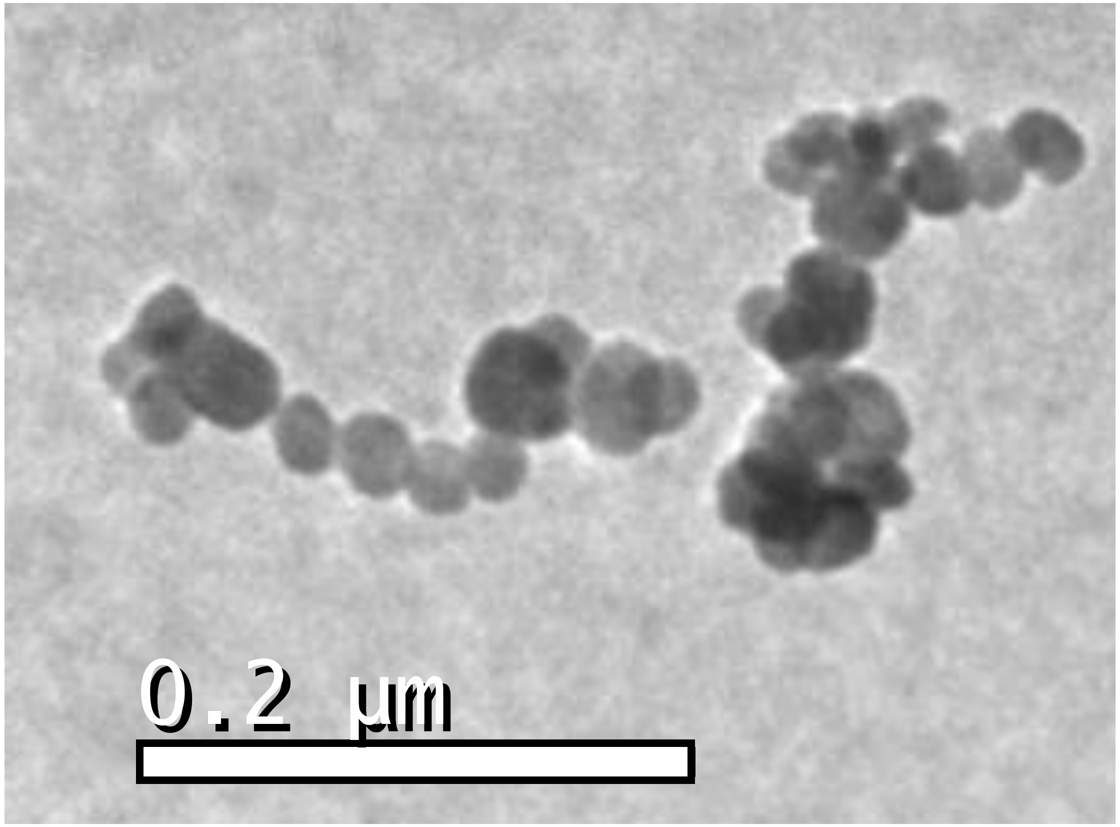

Figure S2. Fe_2O_3 nanoparticles characterisation by HRTEM.

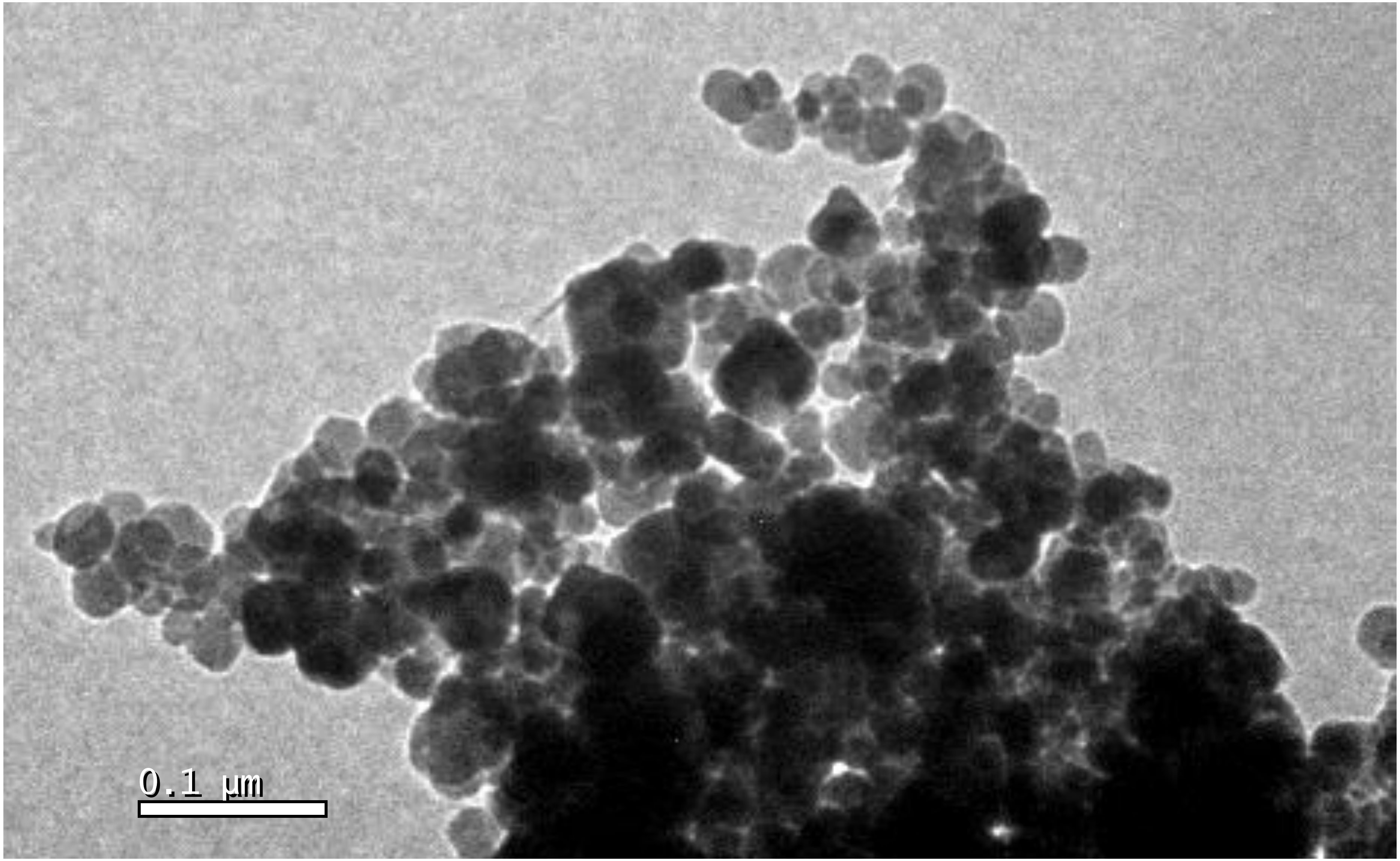

Figure S3. Fe_2O_3 nanoparticles characterisation by HRTEM, corresponding histograms for NPs sizes and electron diffraction pattern.

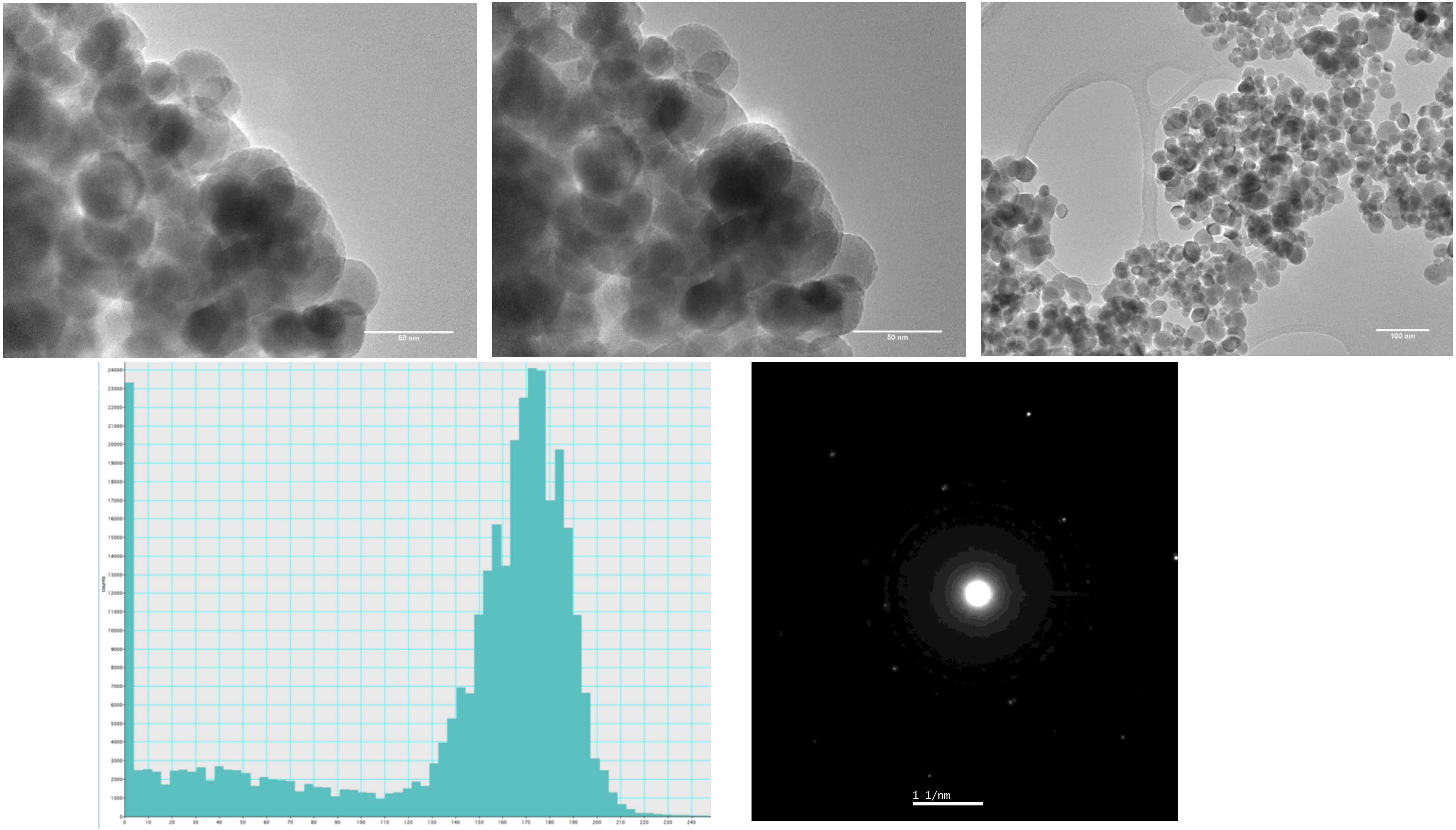

Figure S4. DLS measurements for the Fe_2O_3 NPs dispersed in H_2O (1 mg/ml).

			Size (r.nm):	% Intensity	Width (r.n...
Z-Average (r.nm):	112.1	Peak 1:	126.2	97.8	66.56
PdI:	0.482	Peak 2:	2709	2.2	147.8
Intercept:	0.937	Peak 3:	0.000	0.0	0.000
Result quality	Good				

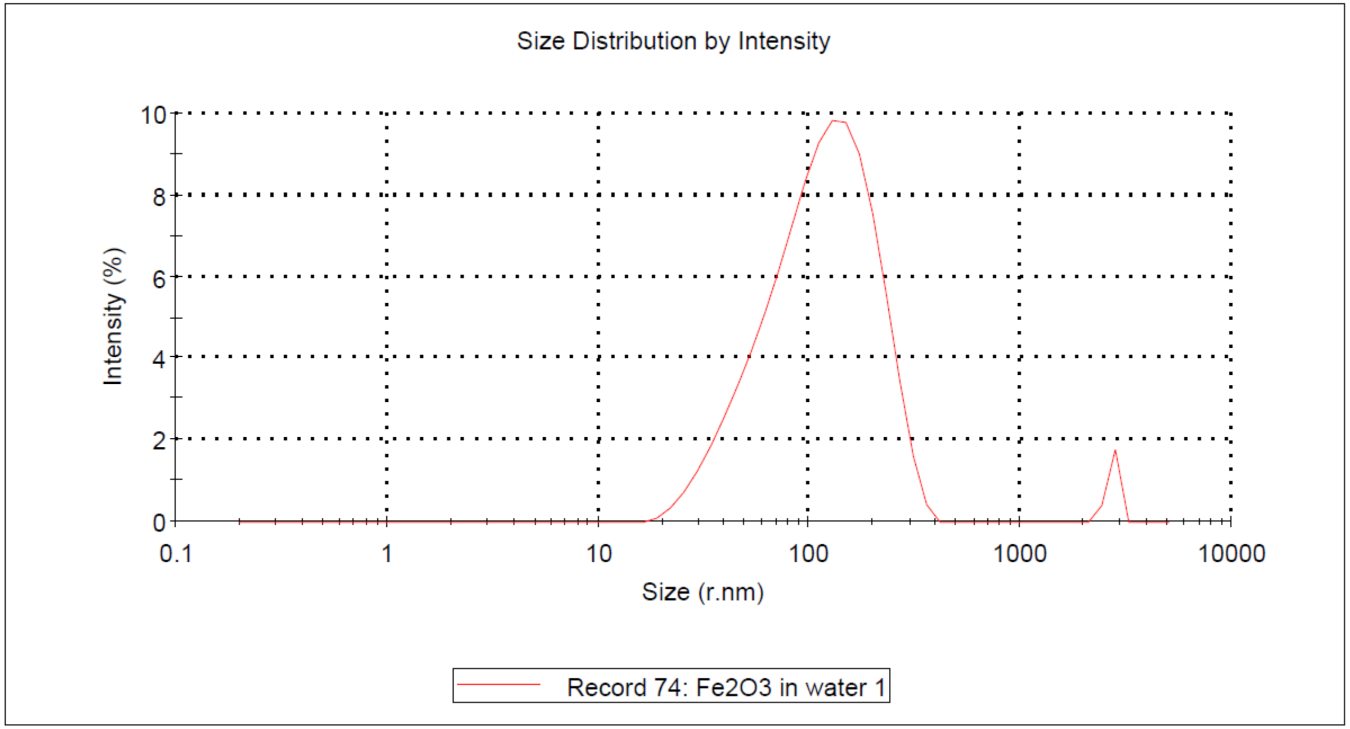

Figures S5. Fe_2O_3 NPs characterisation by TM AFM on a mica surface, at different magnifications.

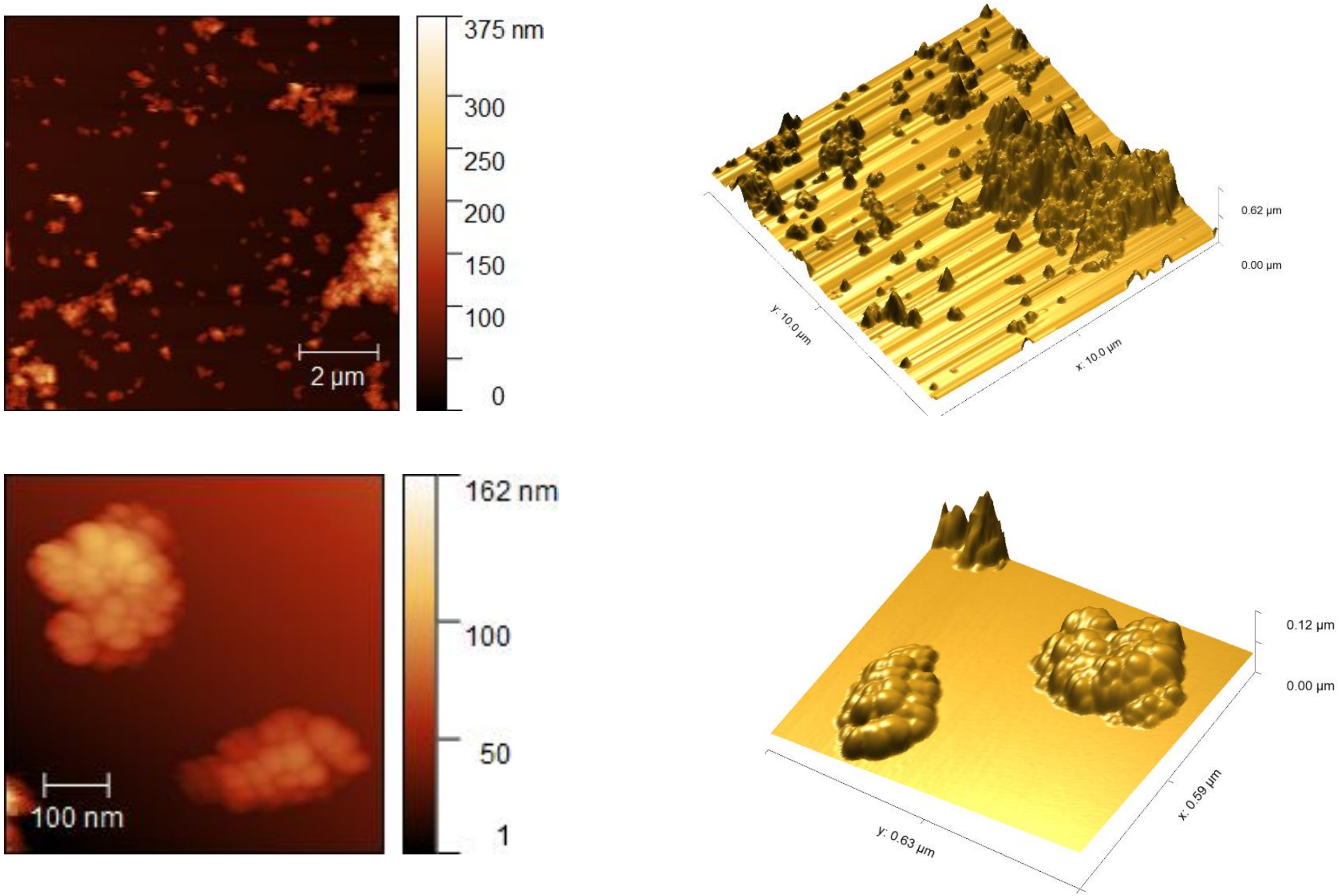

Figure S6. SEM of the graphite oxide support.

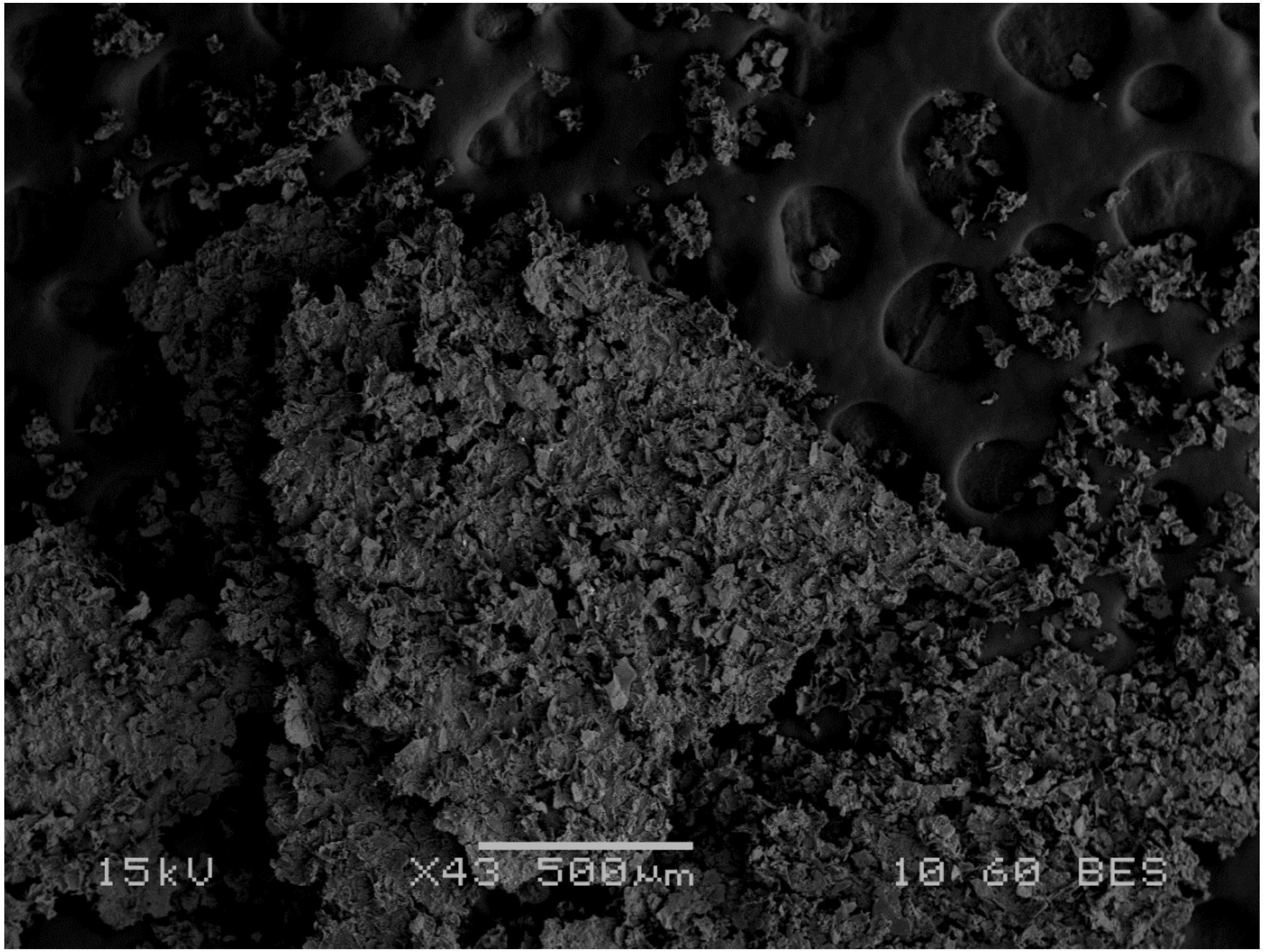

Figure S7. HRTEM of graphite oxide support used, at different magnifications.

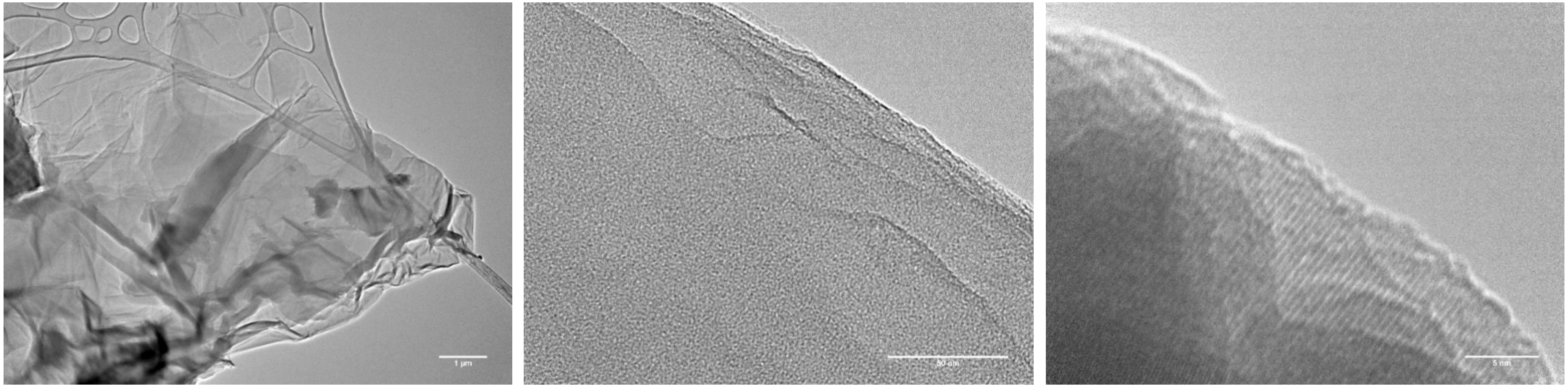

Figure S8. TM AFM of graphite oxide support.

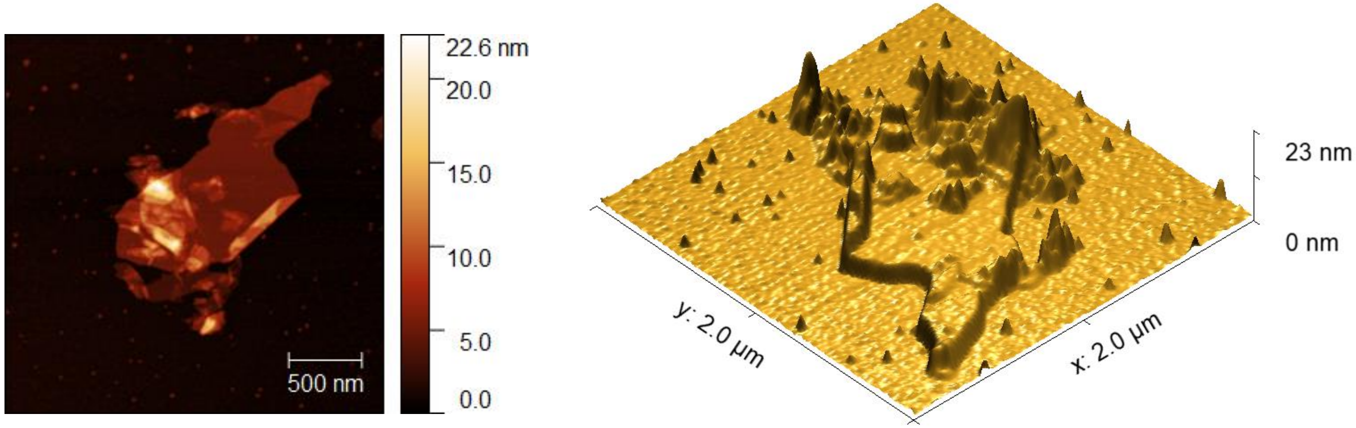

Figure S9. Graphene oxide –bulk characterisation data of batch used as support - by Raman, IR, low res TEM and Electron diffraction.

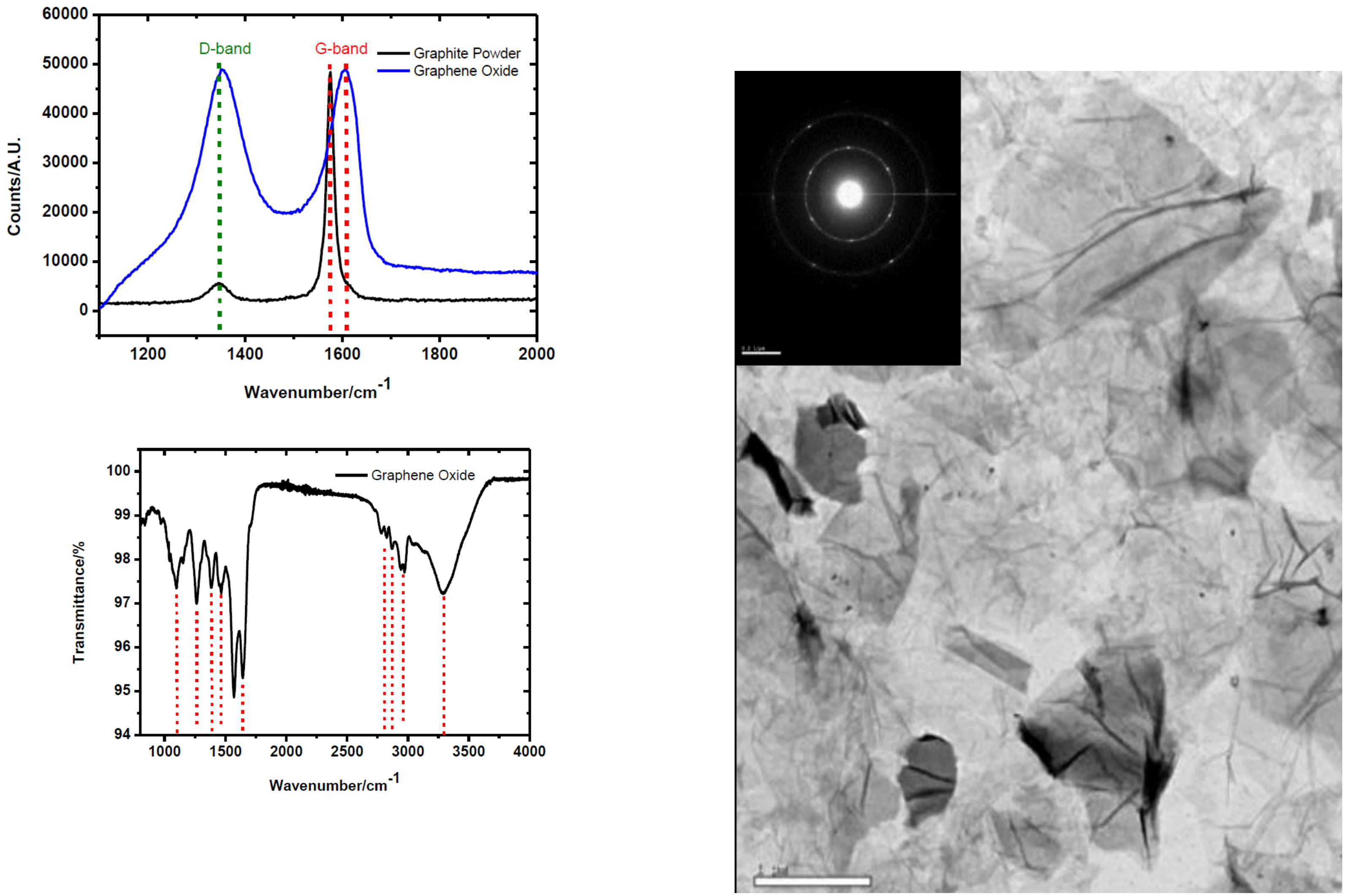

Figure S10. Graphene oxide –bulk characterisation of batch used as support by SEM, corresponding TEM and EDX mapping measurements.

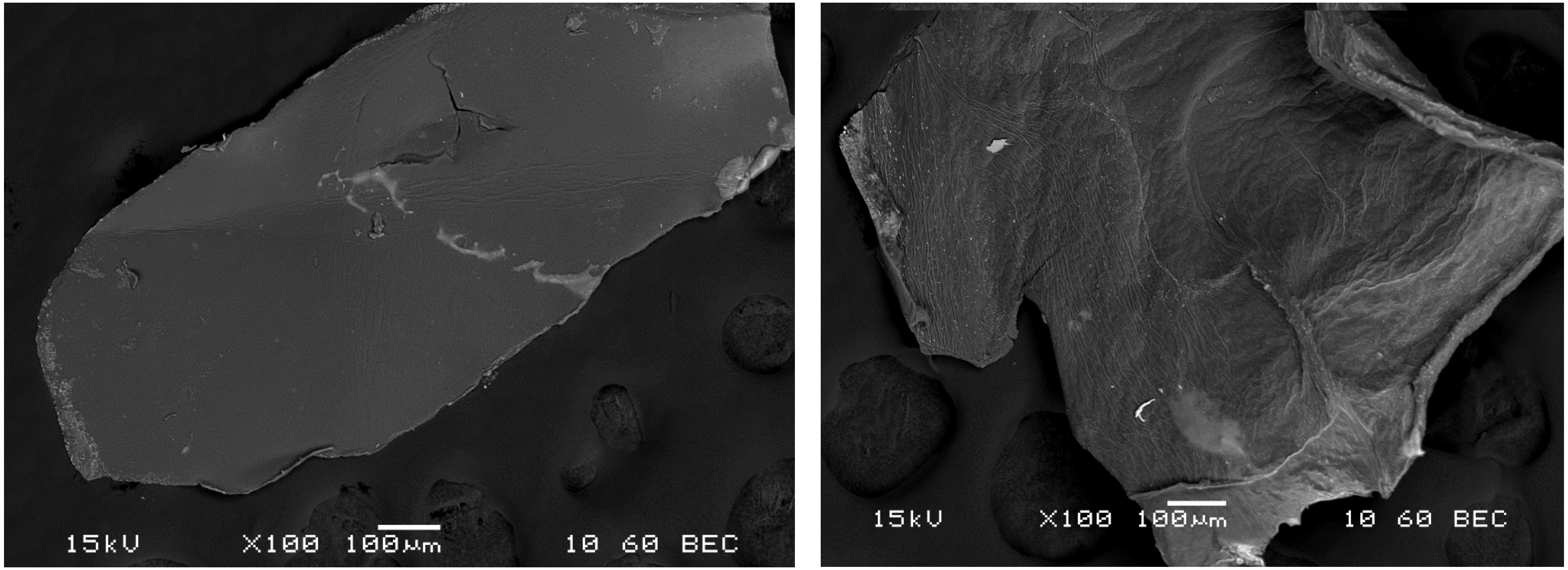

Fig 10 (cont.)

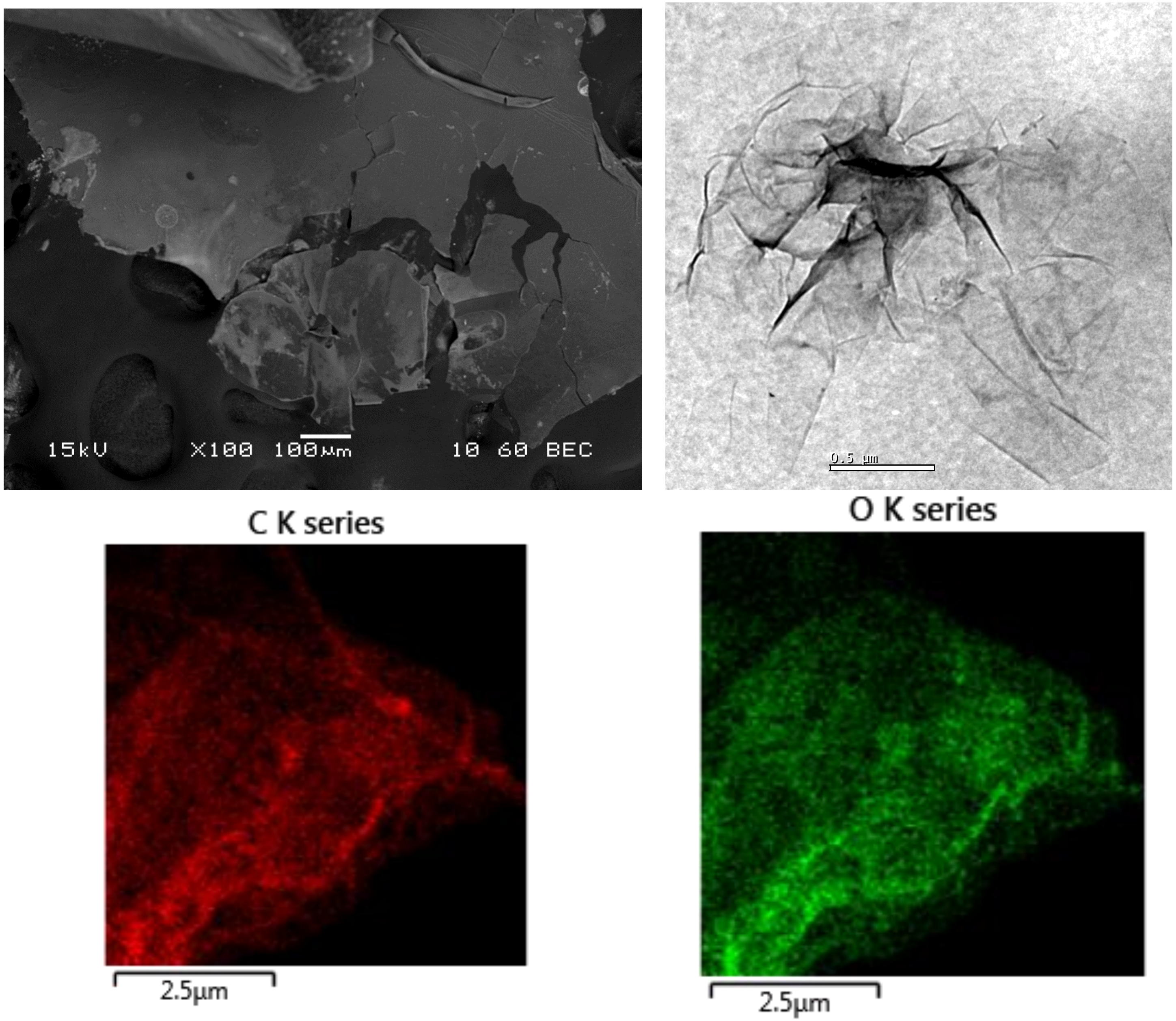

Figure S11. Fe_2O_3-supported onto graphite oxide: TEM and SEM characterisation.

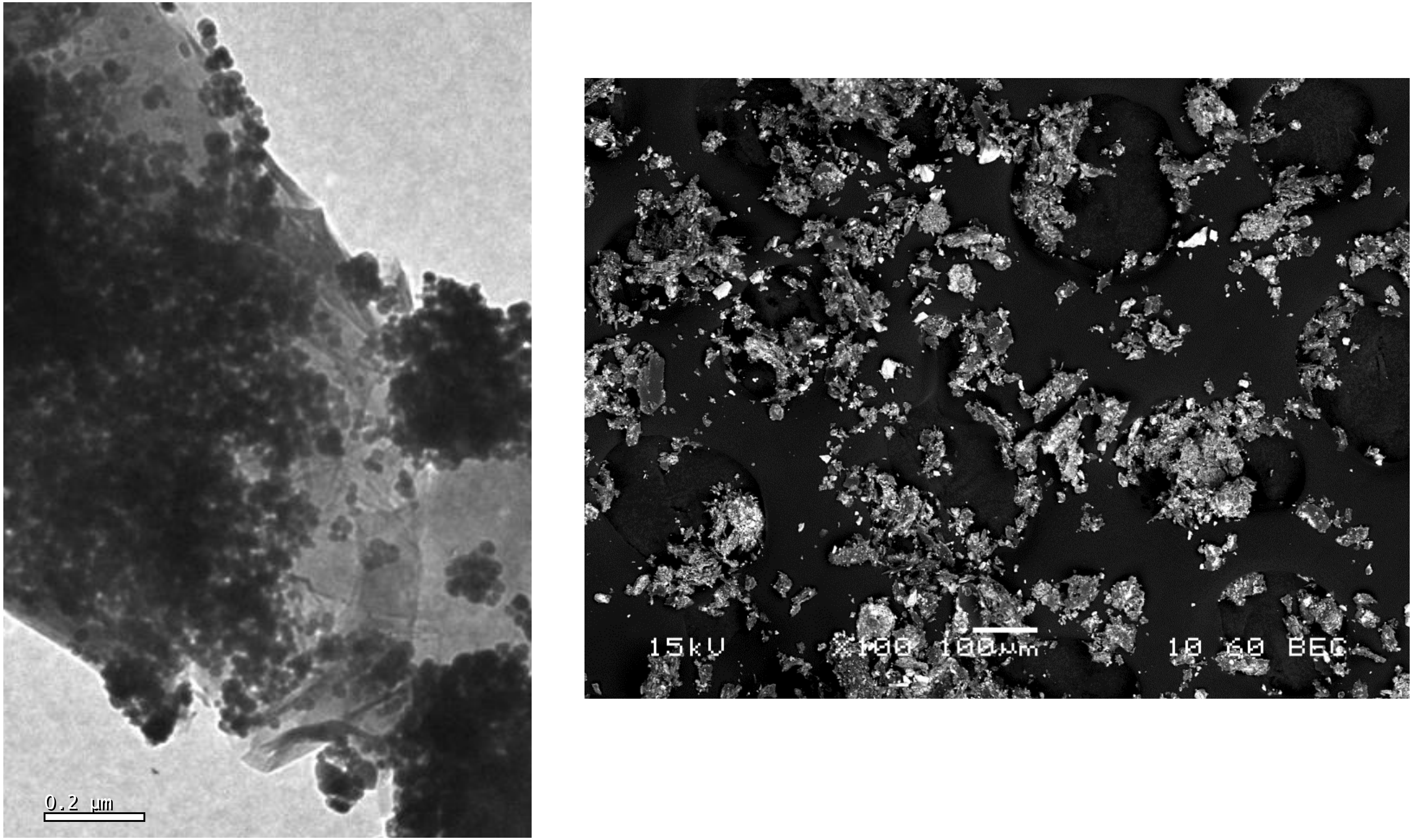

Figure S12. A thin layer graphite oxide decorated with Fe_2O_3 nanoparticles; Fe_2O_3-supported graphite oxide TEM characterisation.

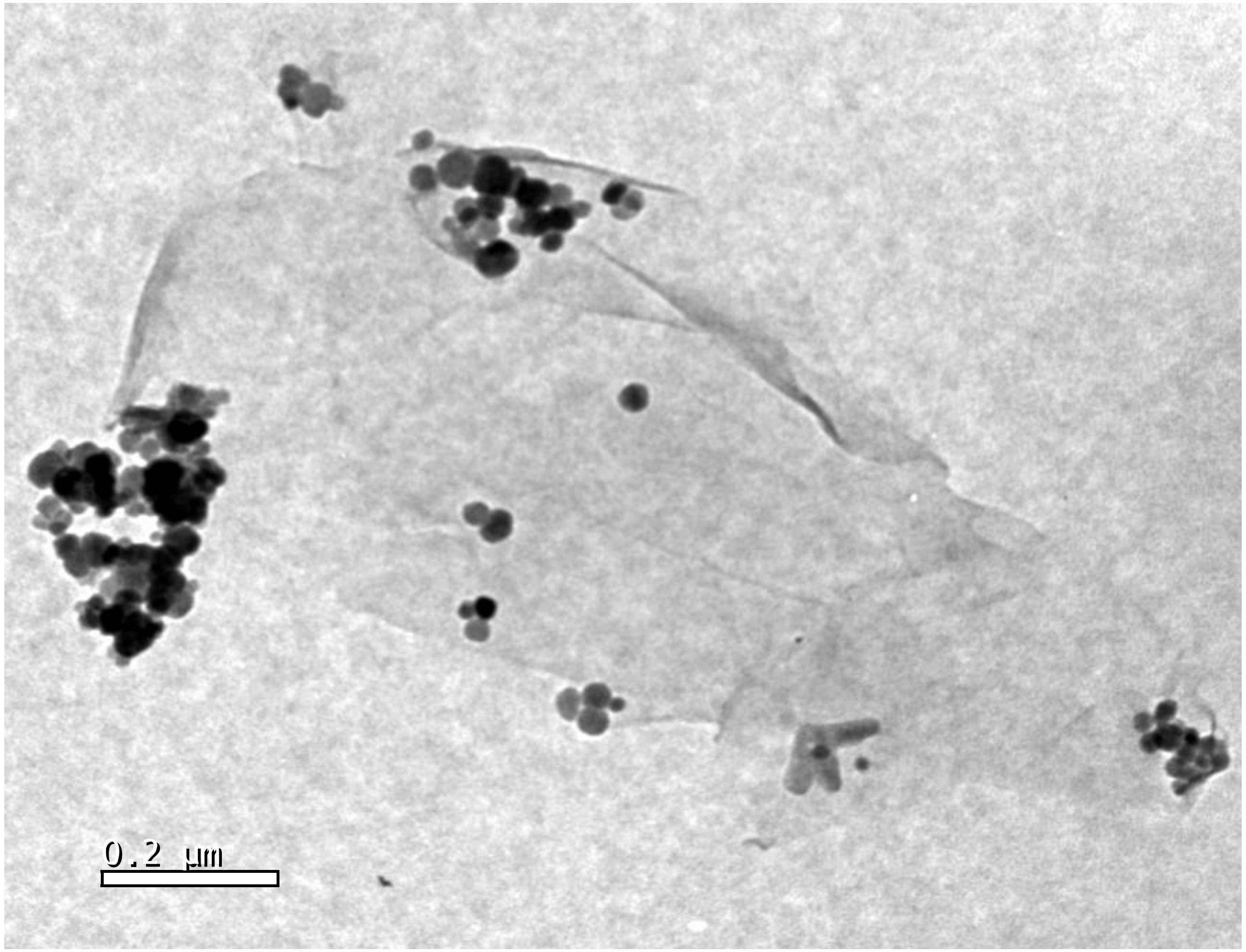

Figure S13. HRTEM images of Fe_2O_3@GO pre-catalysts

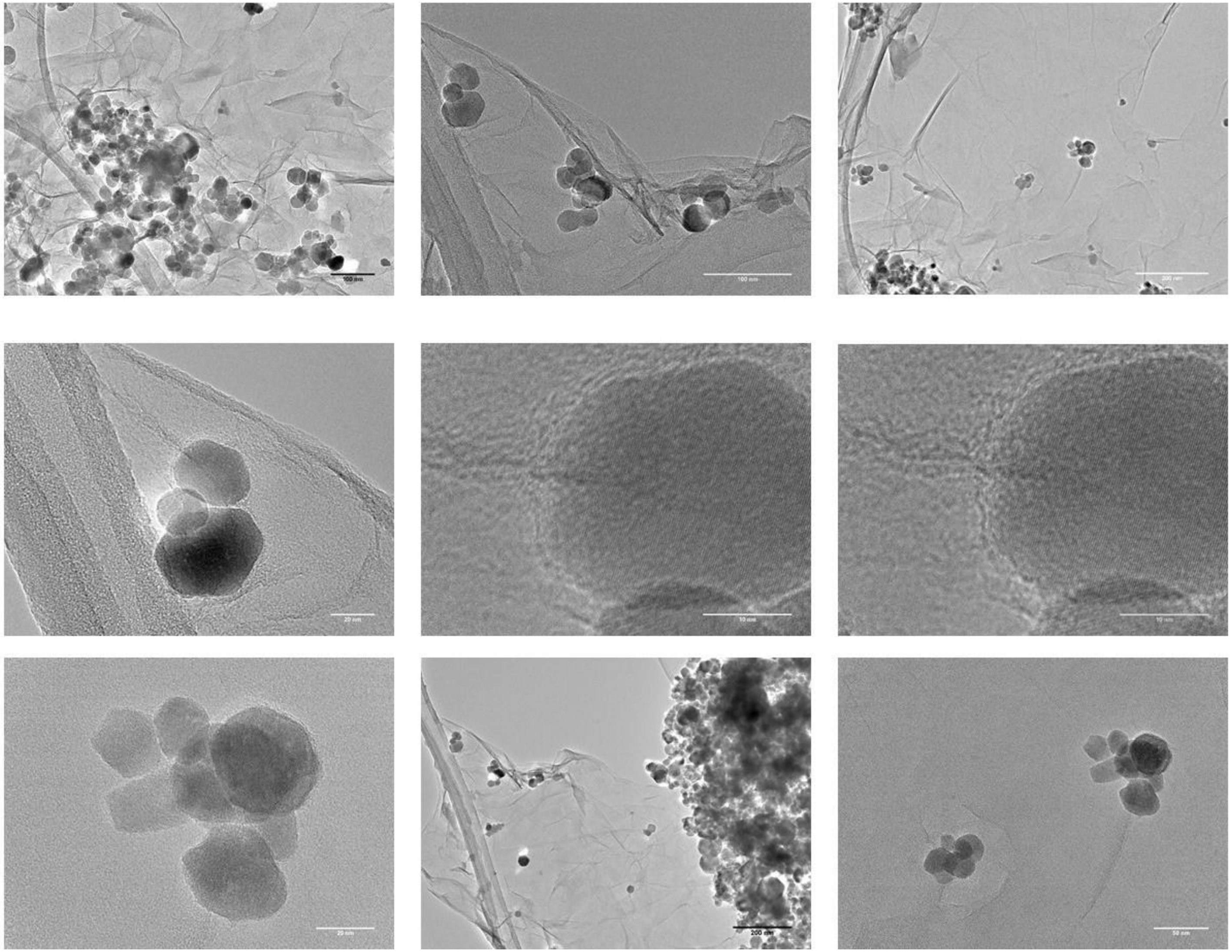

Figure S14. (a) A thin layer graphene oxide decorated with Fe_2O_3 nanoparticles; (b)Fe_2O_3-supported graphite oxide AFM characterisation

(a)

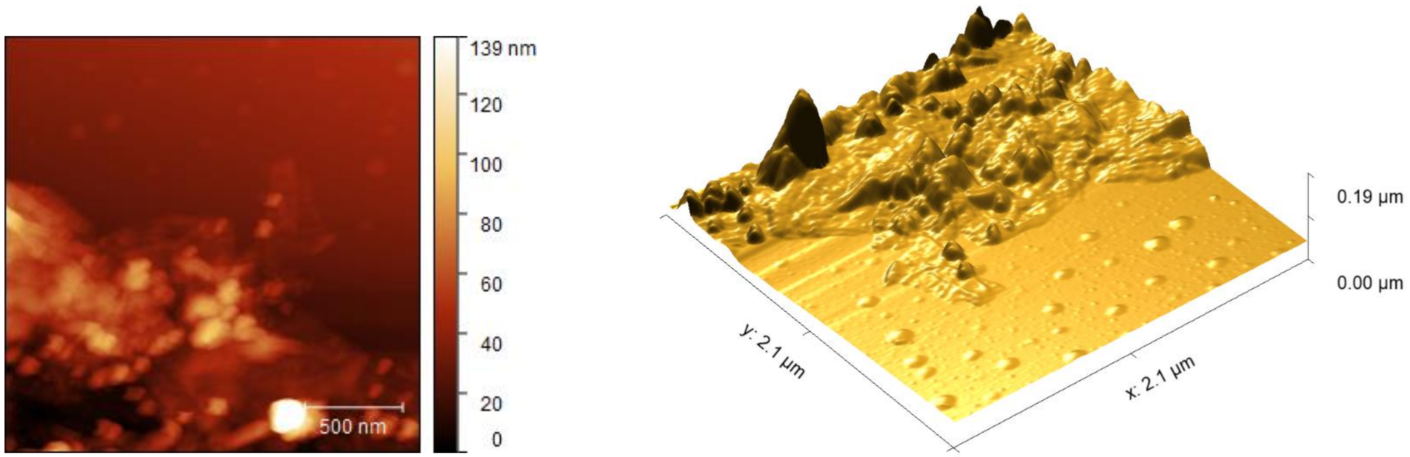

(b)

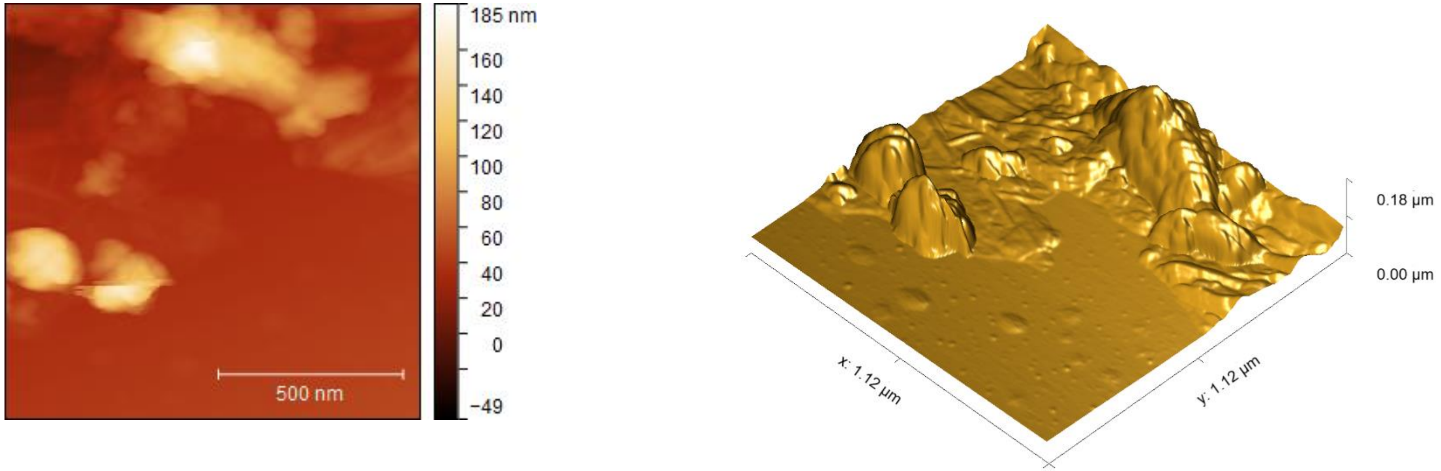

Figure S15. HRTEM images of Pd@Fe_2O_3@GO-a.

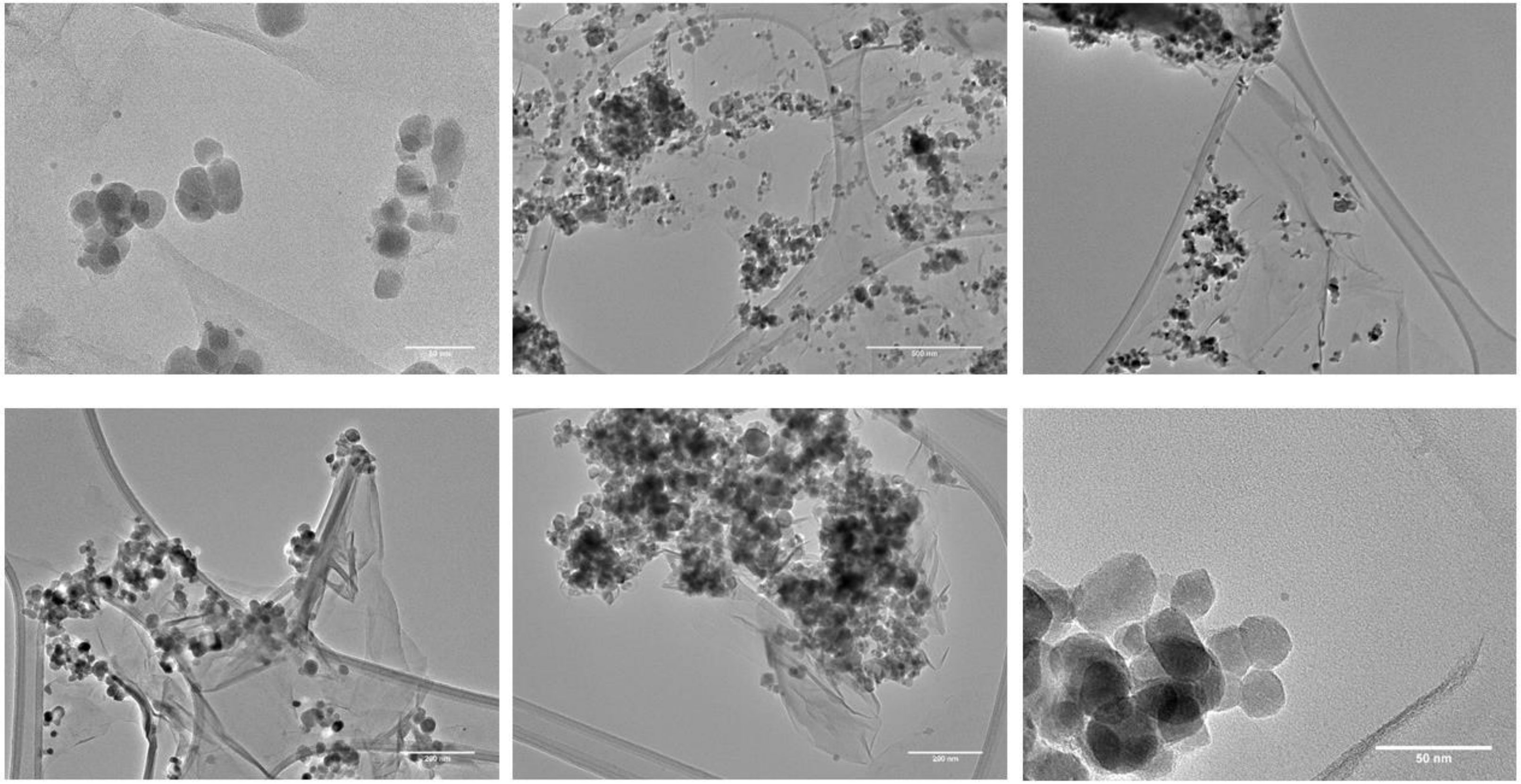

Figure S16. A 3D layered graphite oxide decorated with Pd acetate mixed with Fe_2O_3 nanoparticles (Pd(II)/ Fe_2O_3-supported graphite oxide): TM AFM characterisation

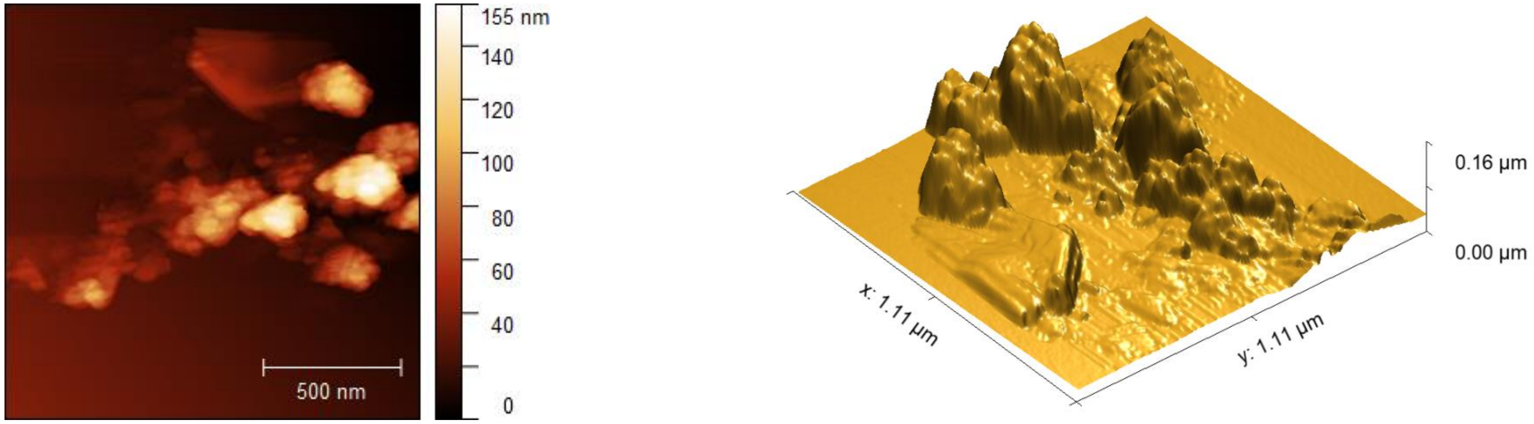

Figure S17. Spectroscopic characterisation by FTIR for the precursors and pre-catalysts used.

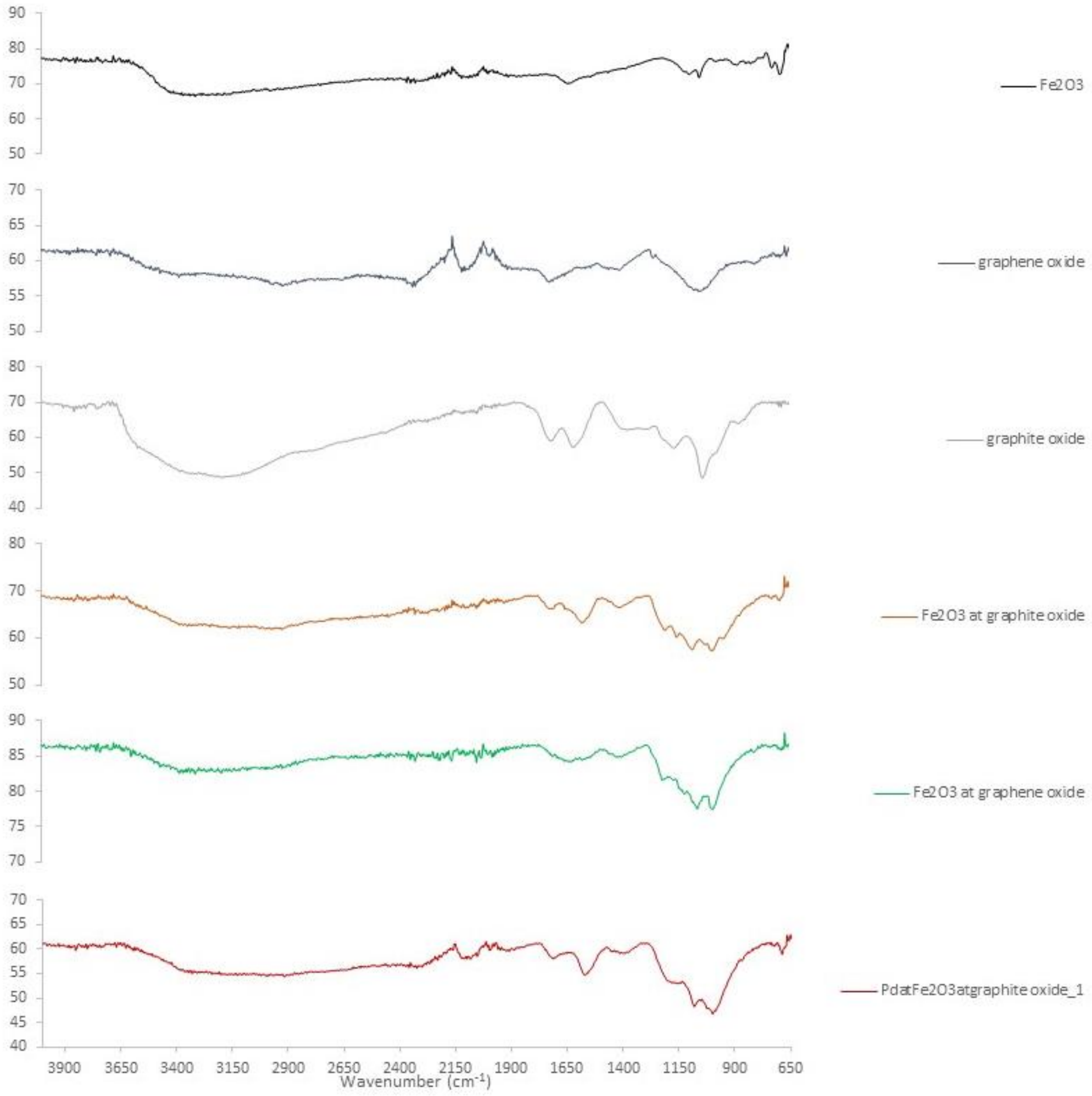

Figure S18. Powder X-ray diffraction patterns for selected precursors and pre-catalysts: (a) γ-Fe_2O_3, (b) Fe_2O_3@Graphene Oxide, (c) $Pd(OAc)_2/Fe_2O_3$@graphite oxide and (d) graphite oxide

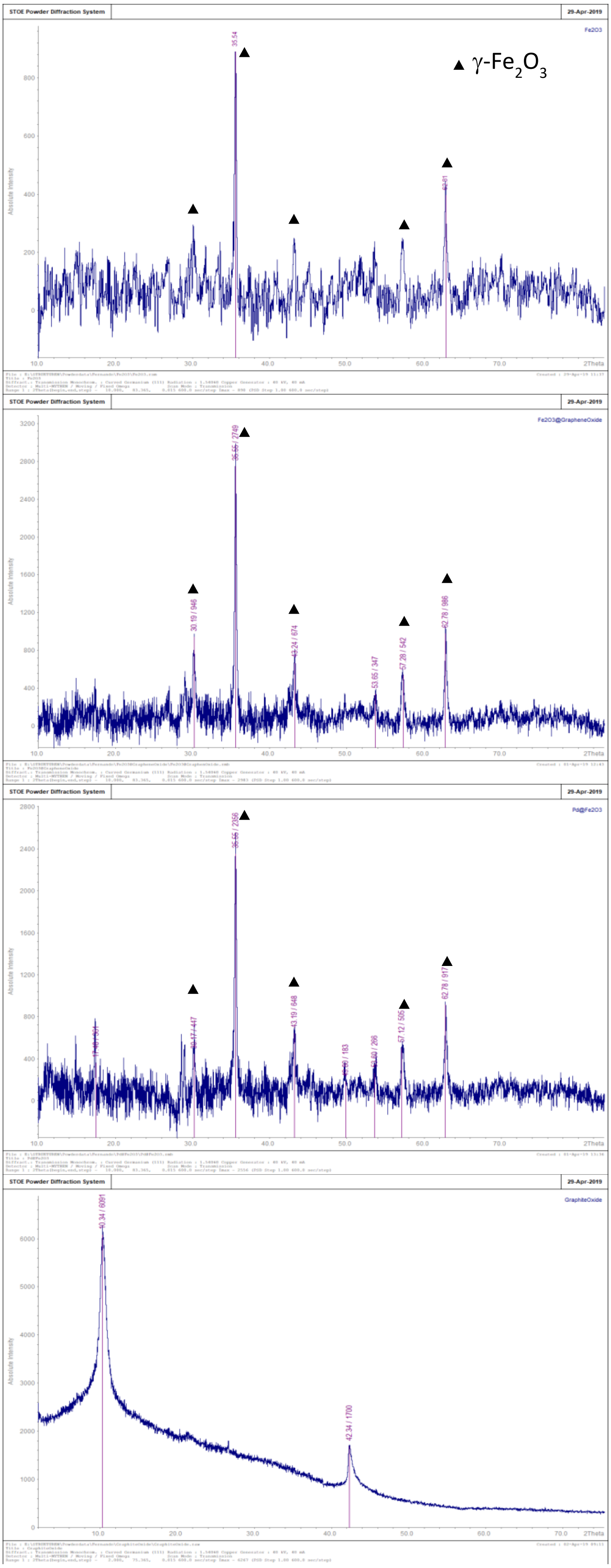

Control experiments:

(a) High temperature annealing and hydrogenation to reduce graphene oxide

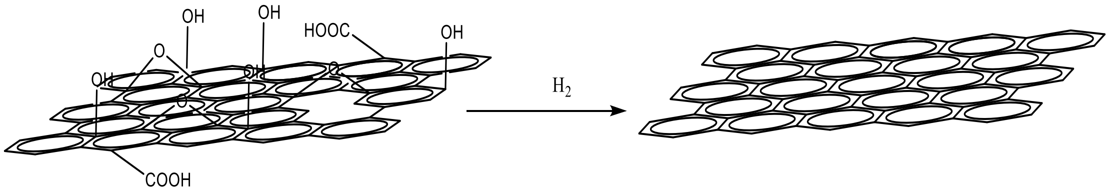

Thermal reduced graphene oxide via an annealing progress has been performed using the adaptation of a reported method.[1-2] It is well known that the rapid increasing high temperature, normally up to around 1050 °C, could reduce the graphene oxide by decomposing oxygen-containing groups. The annealing progress involving reduced graphene oxide was dependent on the annealing temperature: in the measurements of volume electrical conductivity, the 500 °C reduced graphene oxide exhibited only 50 S/cm, while the 700 °C and 1100 °C reduced graphene oxide could reach up to 100 S/cm and 550 S/cm.[3] Since the oxygen etching effects could dramatically increase upon heating to a high temperature, the reduced atmosphere was achieved in the presence of H_2 [4], whereby this was added to rapidly consume the residual oxygen functionalities and to minimise the etching effects.

In a typical experiment, 10 mg of graphene oxide powders were transferred inside the reactor designed as below (consisting of a furnace capable of high temperature heating) using a special designed quartz tube of 9 mm diameter and immobilised with quartz wool at both sides.. The furnace was purged under argon for 2 hours before heating. The increased rate of the temperature was set at 10 °C/min and the furnace was heated up to 1050 °C and kept at this temperature for 2 hours. The whole annealing progress was purged by hydrogen and argon gas (1:9) and then the 100% molecular hydrogen was used as the reductive agent for two hours. After the furnace was cooled down to room temperature, the sample was then collected.

The final yield was 7.4 g (74%) and this was then analysed by HRTEM and Raman spectroscopy. As expected, the high temperature annealing process led to the repair the surface defects by reducing the oxygen groups and carboxylic groups on the surface of the graphene oxide.

From the Raman spectroscopy measurements (873 nm), the graphene oxide exhibited a D band at1347 cm^{-1} and a G band at 1594 cm^{-1}, while the thermal reduced graphene oxide presented a D band at 1350 cm^{-1} and a G band at 1590 cm^{-1}.

For the thermally reduced graphene oxide, the D band and G band both slightly red-shifted, this red-shifted behaviour indicated the surface functional group change, in which case, the removing of the oxygen groups. The I_D/I_G ratio graphene oxide was recorded as 0.91 while the thermal reduced graphene oxide was 0.82: the decrease of I_D/I_G ratio is confirmed the reparation of

the surface defects and disorders in graphene oxide. It can also be found that both D band and G bands of graphene oxide and reduced graphene oxide were rather board which were due to the multi-layered structures in these materials.

The TEM and HRTEM were performed to obtain a direct insight into the structure of thermally reduced graphene oxide layers and compare these with the similar data from the starting materials GO. In the TEM images of thermal reduced graphene oxide, the reduced graphene oxide was still sheet like structures and, compared with graphene oxide, the size of the sheets was around 2 µm. The image also indicated that the sizes of different thermal reduced graphene oxide sheets were varied. Wrinkles-like morphologies also formed on the surface of graphene-type materials. The thermally reduced graphene presented a different contrast, which was due to the different number of carbonaceous layers. After thermal reduction, the obvious defects and disorder has been removed.

Alternative TEM and HRTEM images of thermal reduced graphene oxide from repeated batches are presented below. From the HRTEM images of thermal reduced graphene oxide (with 10 nm scale bar and 5 nm scale bar respectively) the lattice structure can be clearly observed.

Different orientations indicated the trend of sp2 structure and also indicated the multi-layer structure. From the measuring results, the inter-planar spacing of thermal reduced graphene oxide was ca. 0.321 nm, corresponding to the (002) crystallographic planes of graphitic carbon, which is strong evidence of the formation of graphene sheets.

Figure S19. Powder X-ray diffraction patterns for graphene oxide and a comparison with the corresponding data of the thermally reduced graphene oxide [*Adapted from J. Tyson, PhD Thesis 2016, University of Bath*].

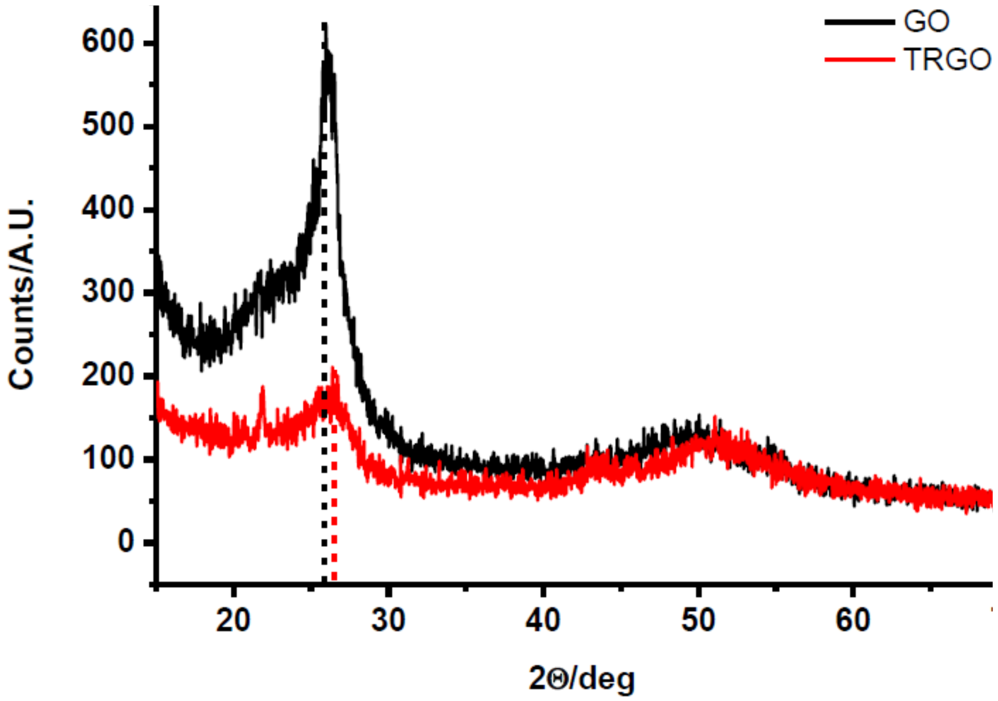

Figure S20. Spectroscopic characterization by Raman spectroscopy for graphene oxide and high temperature thermally reduced graphene oxide.

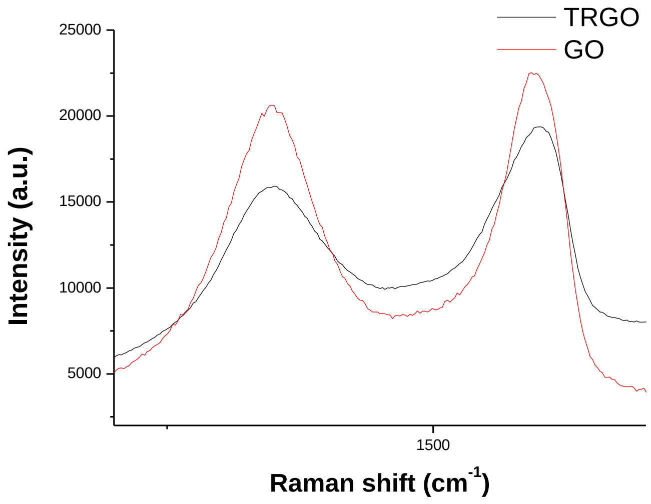

Figure S21. Solid state investigation by TEM: TEM image of high temperature thermally reduced graphene oxide, scale bar = 1 μm **(b)** magnified TEM image of thermal reduced graphene oxide, scale bar = 200 nm **(c)** HRTEM image of thermal reduced graphene oxide, scale bar = 10 nm and **(d)** HRTEM image of thermal reduced graphene oxide, scale bar = 5 nm.

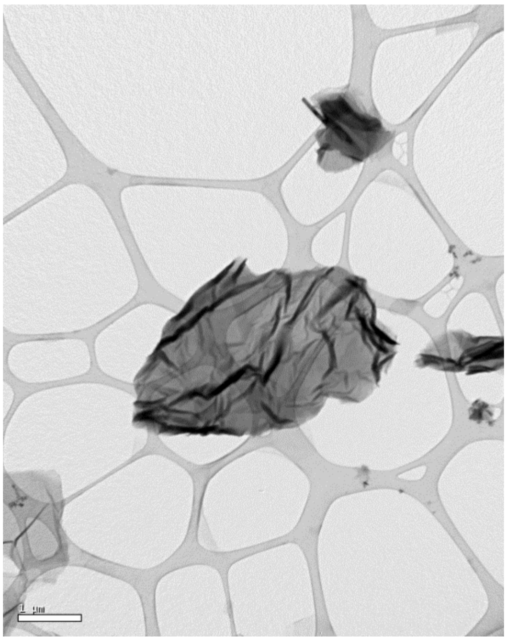

(a)

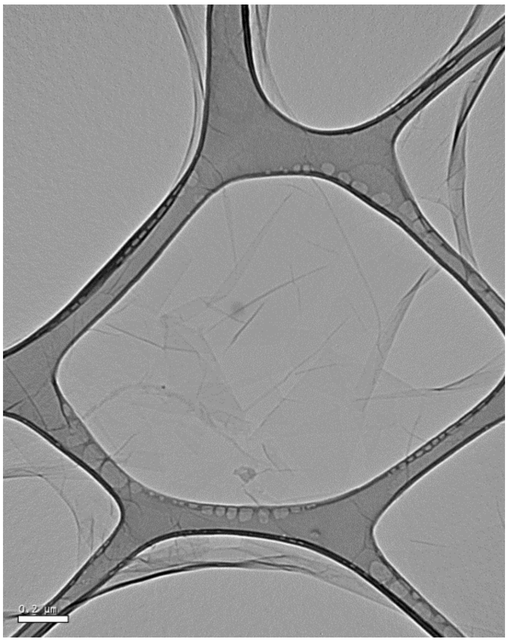

(b)

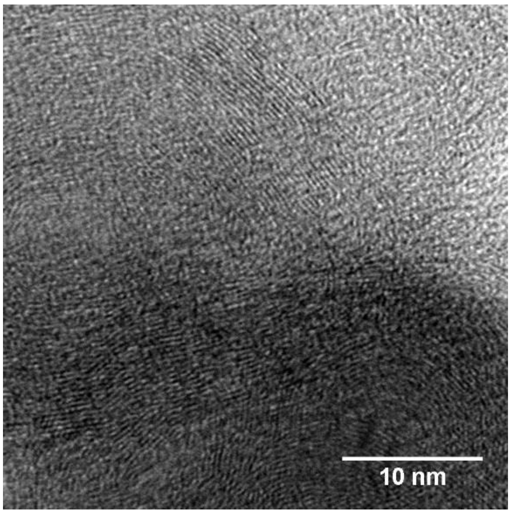

(c)

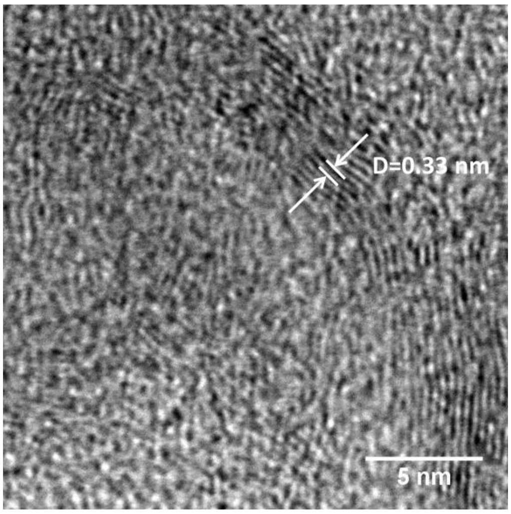

(d)

Figure S22 Additional TEM images of thermally reduced graphene oxide emerging from high temperature reduction.

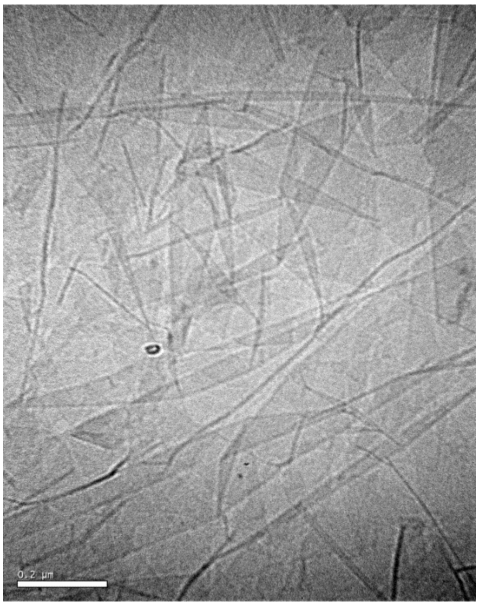

Figure S23 HRTEM images of thermal reduced graphene oxide .

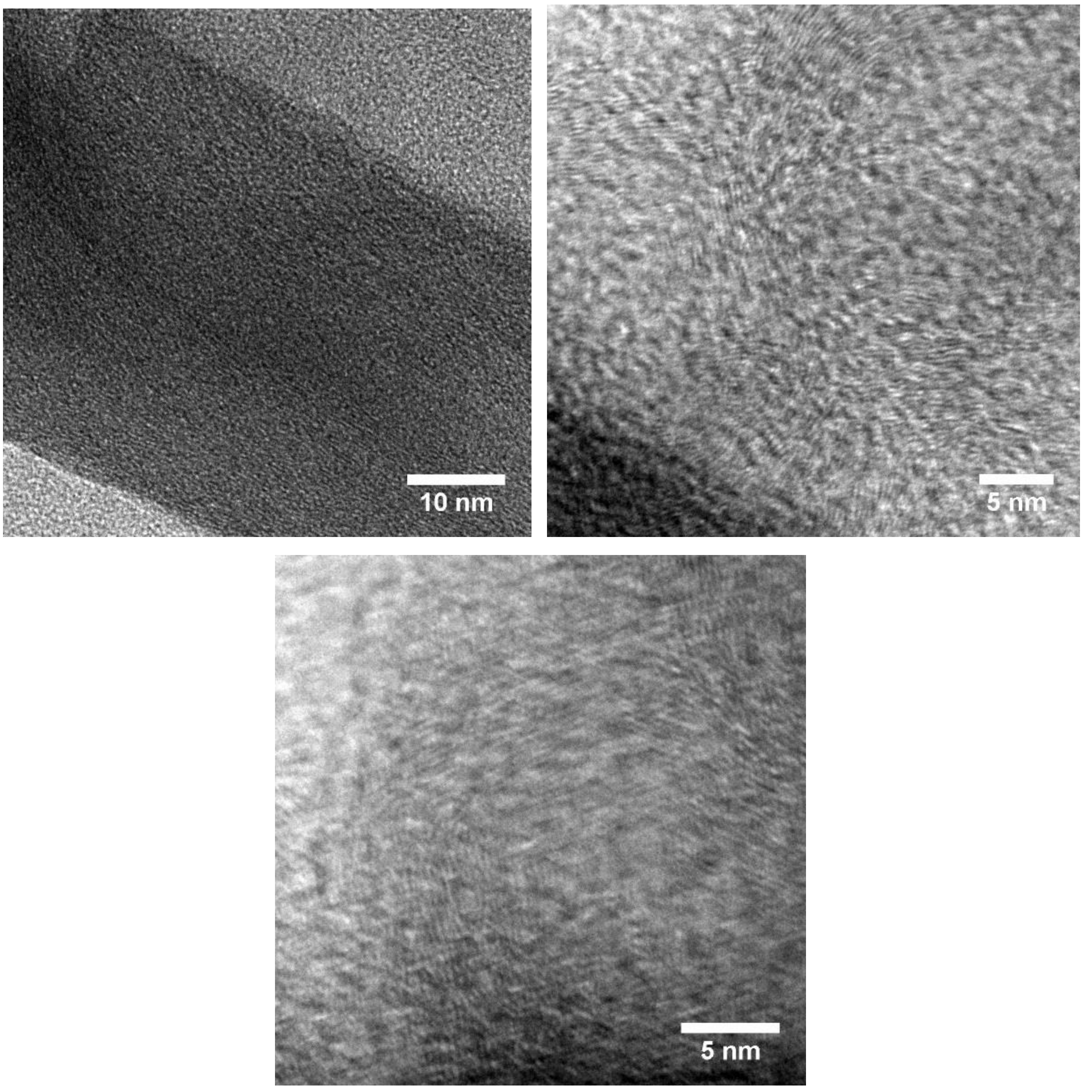

Control Experiments (B): Direct synthesis of magnetic Fe_3O_4 nanoparticles

A mixture of $FeCl_3 \cdot 6H_2O$ (5.4 g, 20 mMol) and $FeCl_2 \cdot 4H_2O$ (2.0 g, 10 mMol) was dissolved under N_2 in an HCl solution (10.3 mMol in 25 ml H_2O. This as-prepared solution was added dropwise to 250 ml of a deoxygenated 1.5 M NH_4OH with vigorous mechanical stirring.

The black precipitate was isolated from the solvent *via* magnetic decantation. The washing–decantation procedure was repeated three times, followed by washing twice with a tetramethylammonium hydroxide solution (100 ml of 0.1 M TMAOH). The suspension was precipitated with acetone and methanol, and the precipitate dried in vacuum overnight and over $CaCl_2$. Magnetic nanoparticles prepared using this procedure were very easy to separate using a standard permanent magnet (surface magnetization 0.3 T, **MMG MagDev, UK**). To prevent aggregation these magnetic NPs (with dimensions recorded by DLS in H_2O ranging between 10-40 nm) were either stored as a 1 mg/ml dispersion in Me_2NOH, oleic acid or coated with a SiO_2 matrix using a micro emulsion protocol. [5]

Figure S24: TEM micrograph of as-synthesised Fe_3O_4 nanoparticles. Scalebar: 100 nm.

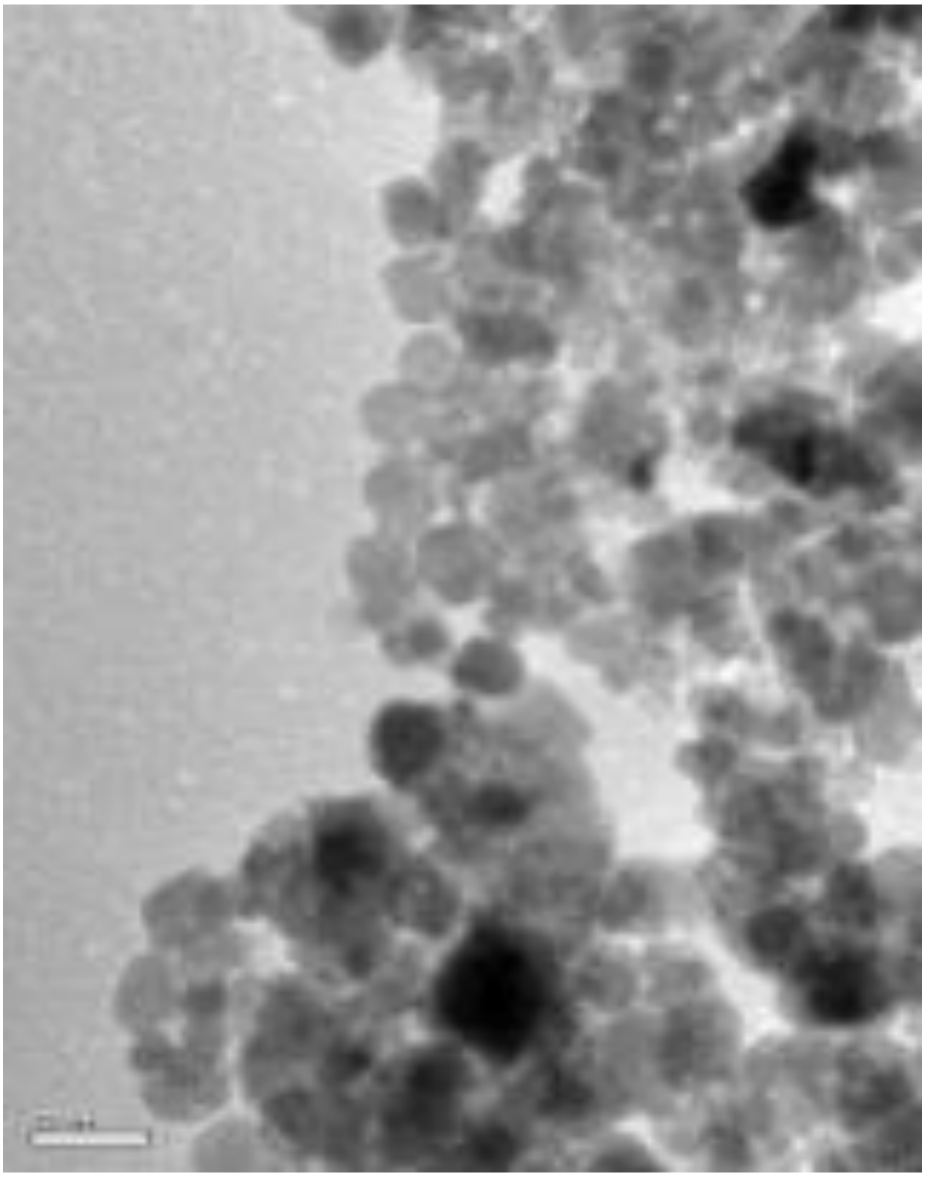

Figure S25: Powder XRD patterns for the as-prepared Fe_3O_4 showing characteristic diffraction pattern of the magnetite phase (ICDD file no. 86–2368)

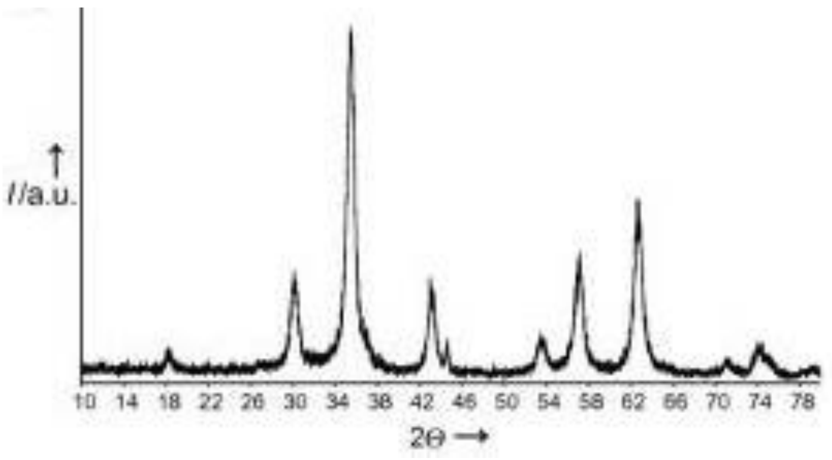

Catalyst testing:

The experiment was performed for each iron-based graphene-supported pre-catalyst using our purpose built stainless steel packed-bed reactor (1/2" diameter x 12 cm length) that can be heated to a variety of temperatures.

The pre-catalysts (with masses given in Experimental Section) were first activated (reduced) under a pure flow of H_2 (50 sccm) at 300 ºC for 2 hours under atmospheric pressure and used in situ thereafter. For typical carbon dioxide activation experiments, CO_2 (2 sccm) and H_2 (6 sccm) were passed over in flow over the activated nano-catalysts at 4 different temperatures: 300, 335, 370 and 405 ºC to allow a comparison of catalytic activities and selectivity, over 65 h periods. The product gases were analysed using gas chromatography mass spectrometry (GCMS) on gas samples collected manually from the exhaust gases of the reactor using an air-tight gas syringe. Typically 30 ml of gas is sampled using a gas syringe and injected into an Agilent 7890A GCMS with a HP-PLOT/Q, 30 m long 0.530 mm diameter column. At the start of the experiment the GC-MS was calibrated with a BOC special gas with each gas composition 1 % v/v CH_4, C_2H_6, C_3H_6, C_3H_8, C_4H_{10}, CO, CO_2, with N_2 carrier gas. The composition of the outlet gases was analysed using GC-MS and the molar composition was calculated from the peak area and response factors calculated from the calibration gases. The carbon mass balance was estimated as follows: the total volume and composition of the injected gases was calculated per hour. In all cases the mass balance was found to be satisfactory and within the range of experimental error (10%).

Figure S26. GC MS-coupled Catalysis Rig Diagram Sample was analysed by Agilent Technologies, 7890A containing three detectors: i) Mass Spectrometer Agilent Technologies 5975 C inert MSD with triple axis detector ii) Flame Ionisation Detector iii) Thermal Conductivity Sensor GC System

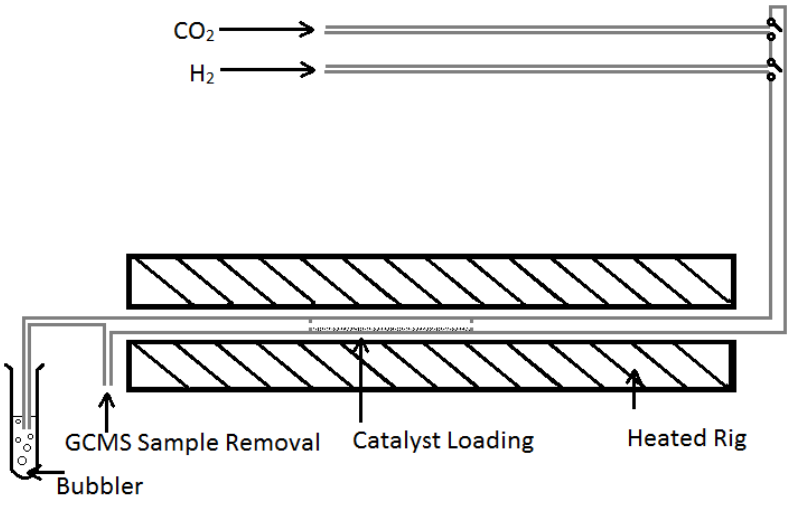

Equations used to calculate Carbon balance, conversion and selectivity are given below:

$$Balance = \frac{Total\ moles\ of\ carbon\ in\ product\ stream/hr}{Total\ moles\ of\ carbon\ added\ per/hr}\ X\ 100\%$$

$$Conversion = \frac{Moles\ of\ CO_{2\ in} - Moles\ of\ CO_{2\ out}}{Moles\ of\ CO_{2\ in}}$$

$$Selectivity\ of\ Product\ x = \frac{Moles\ of\ carbon\ in\ product\ x}{Moles\ of\ CO_2\ converted}$$

Figure S27 Hydrocarbon selectivity for Fe@GO; Fe@GO-a and Pd-Fe@GO-2 pre-catalysts at 405ºC, over 40 minutes. Note: 0.1 g of iron oxide functionalised graphene oxide was hydrogenated for 2 hours at 300 ºC and then ran through the catalysis rig. Samples of gas were taken and analysed by GC MS after each of the catalyst cycle of 40 min. Hydrocarbons as well as aromatic products including benzene were observed suggesting a breakdown of the catalysts above 370 ºC.

Methane (1)	Ethylene (2)	Ethane (3)	Propene (4)	Propane (5)	C4+ (6)

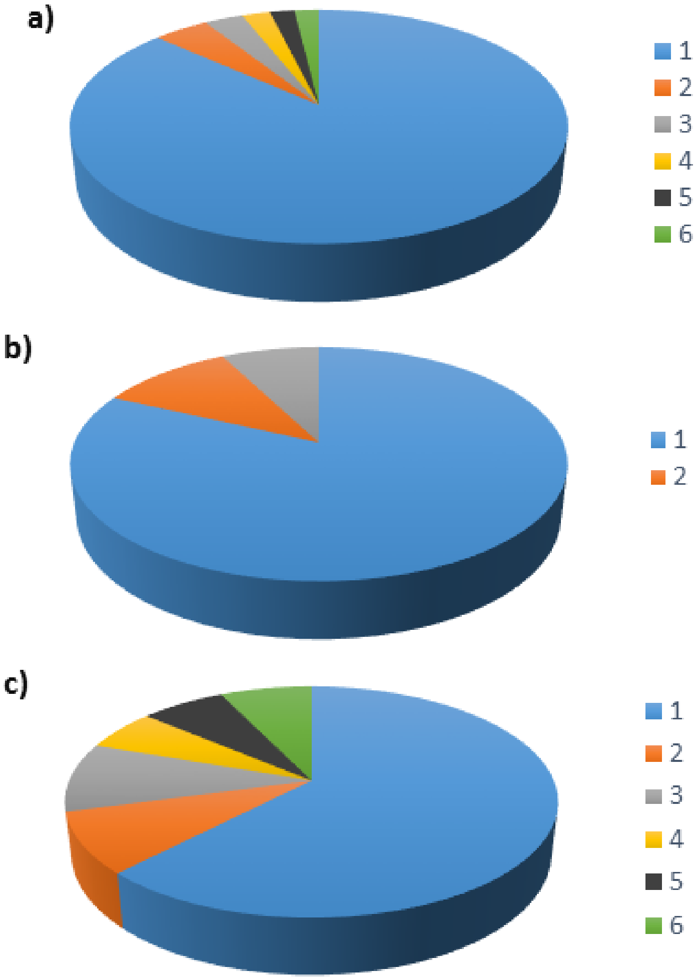

Figure S28. A comparison with Nanostructured Iron Catalysis deposited on Silica (A) and deposited on multiwall carbon nanotubes (B) under similar temperature and pressure conditions, albeit tested over 24 h

Data adapted from Reference 7, whereby work was carried out in the same catalytic rig.

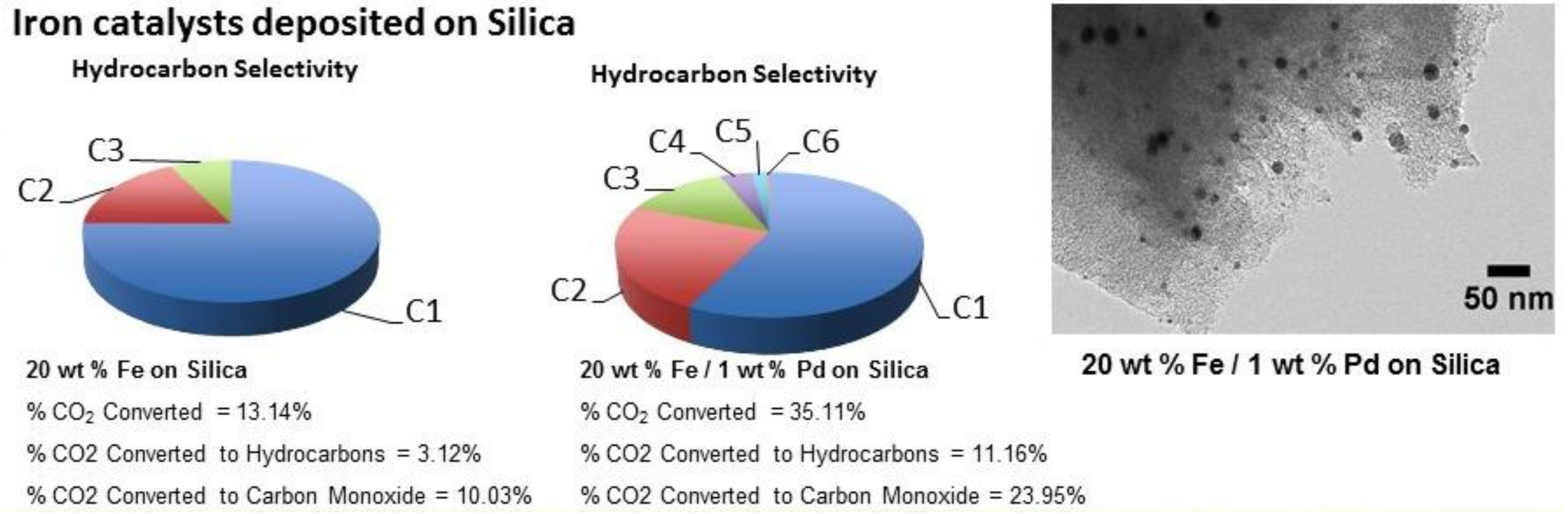

Figure S29. A comparison with Nanostructured Iron Catalysis deposited on Silica (A) and deposited on multiwall carbon nanotubes (B) under similar temperature and pressure conditions, albeit tested over 24 h

Data adapted from Reference 8., whereby work was carried out in the same catalytic rig.

Iron catalysts deposited on CNTs

**Conversion of CO_2**	
**Total Conversion**	34.5 %
**Carbon Monoxide**	15.1 %
**To Hydrocarbons**	19.4 %

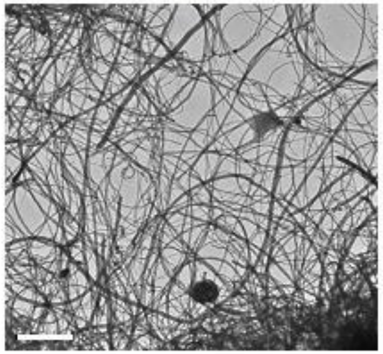

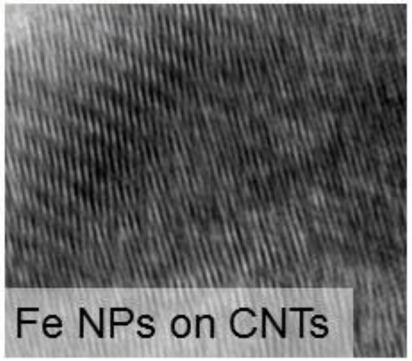

Table S1. Calculated % atomic concentration in XPS

Sample	Fe	C	O	S*	F*
GO-a	-	66.78	31.77	1.45	-
FeGO-a	3.41	59.81	36.48	0.30	-
[FeGO-a] (H_2-Reduced)	1.41	77.51	19.93	1.16	-
PdFeGO-a	1.81	63.90	26.00	1.47	6.82
[PdFeGO-a] (H_2-Reduced)	0.77	75.92	19.28	0.56	3.46

*The presence of sulphur over the surface of all the GO-a samples is due to the Hummer's treatments carried out to obtain the GO-a and GO samples. PTFE contaminants coming from PTFE taps of Schlenk tubes used to remove the toluene under reduced pressure. These PTFE contaminants do not affect the activity of Pd promoted FT catalyst.

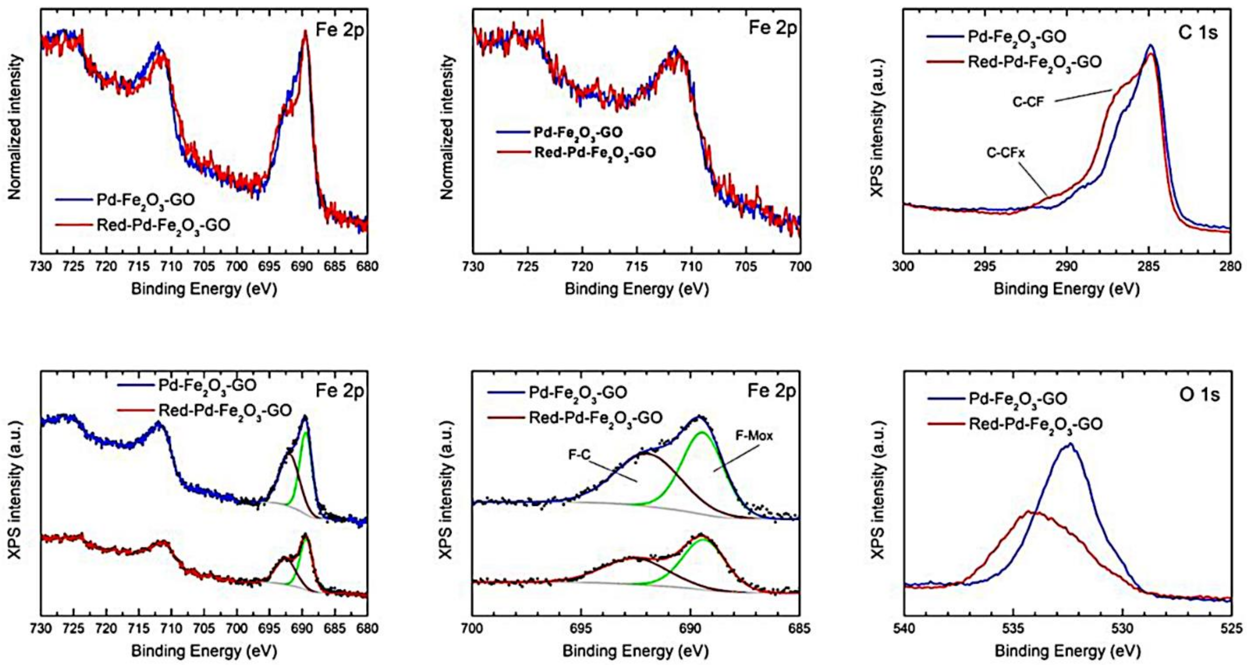

Figure S30. High-resolution XPS spectra corresponding to the Fe2p, C1s and O1s core levels and FeGO-a, Reduced-FeGO-a, PdFeGO-a and reduced PdFeGO-a samples.
In the samples with Pd it is also detected the presence of F, in particular C-F groups. This signal is due to PTFE contaminants coming from PTFE taps of Schlenk tubes to remove the toluene under reduced pressure, during the synthesis of the PdFeGO-a and PdFeGO samples. It has been checked that these PTFE contaminants do not affect the activity of Pd promoted FT catalyst.

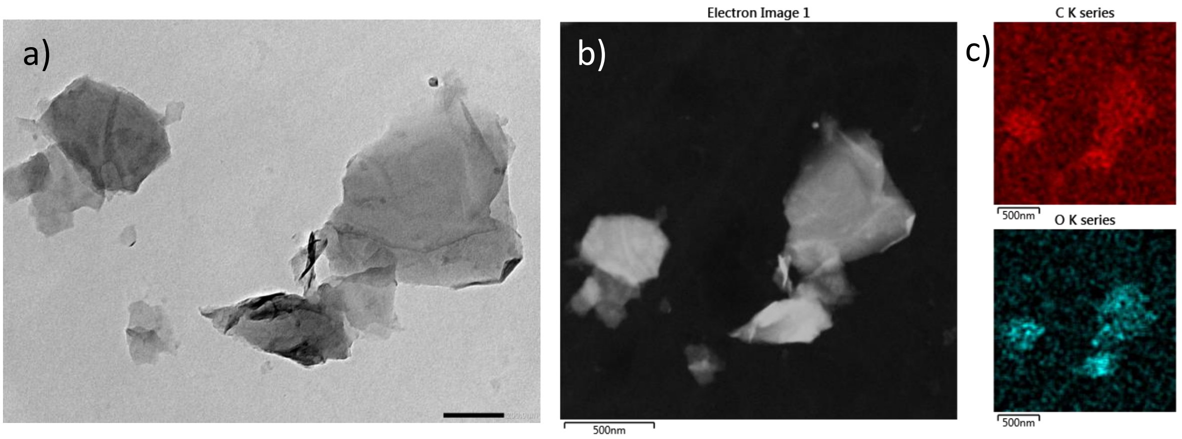

Figure S31. Electron microscopy analysis of [FeGO-a] (H_2 reduced). a) TEM image, scale bar: 200 nm; b) SEM image, scale bar: 500 nm; c) corresponding EDX mapping analysis.

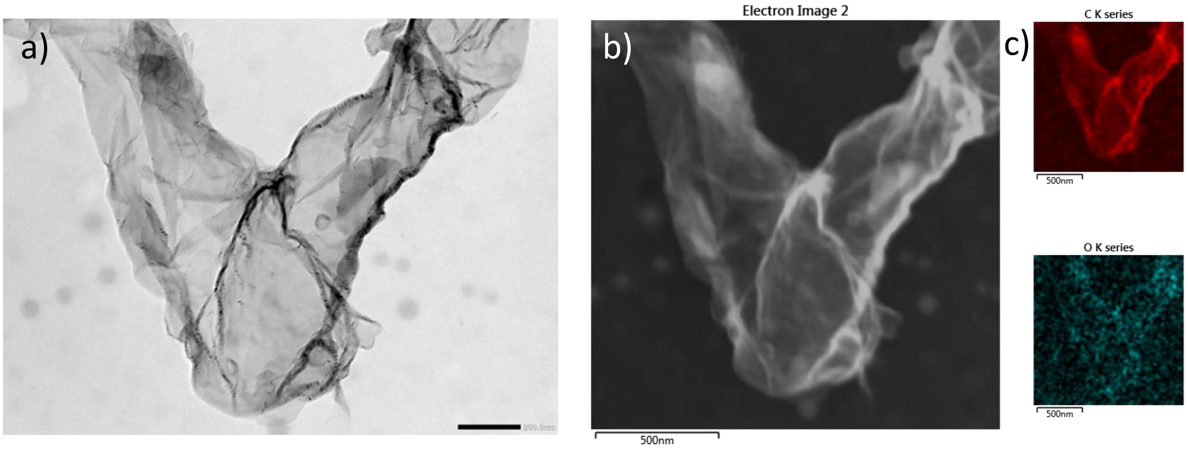

Figure S32. Electron microscopy analysis of [FeGO] (H_2 reduced). a) TEM image, scale bar: 200 nm; b) SEM image, scale bar: 500 nm; c) corresponding EDX mapping analysis.

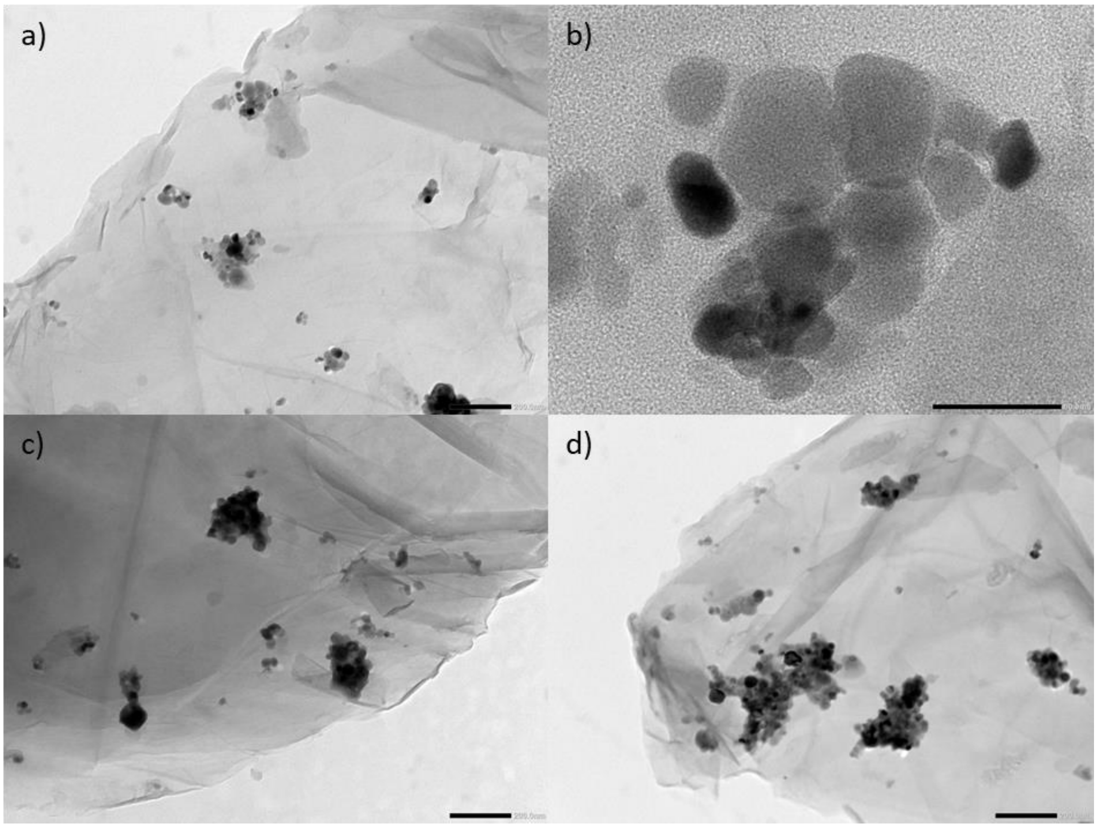

Figure S33. HRTEM images of [FeGO-a] (H_2 reduced) at different magnifications. a) Scale bar: 200 nm; b) scale bar: 50 nm; c), d) scale bar: 200 nm.

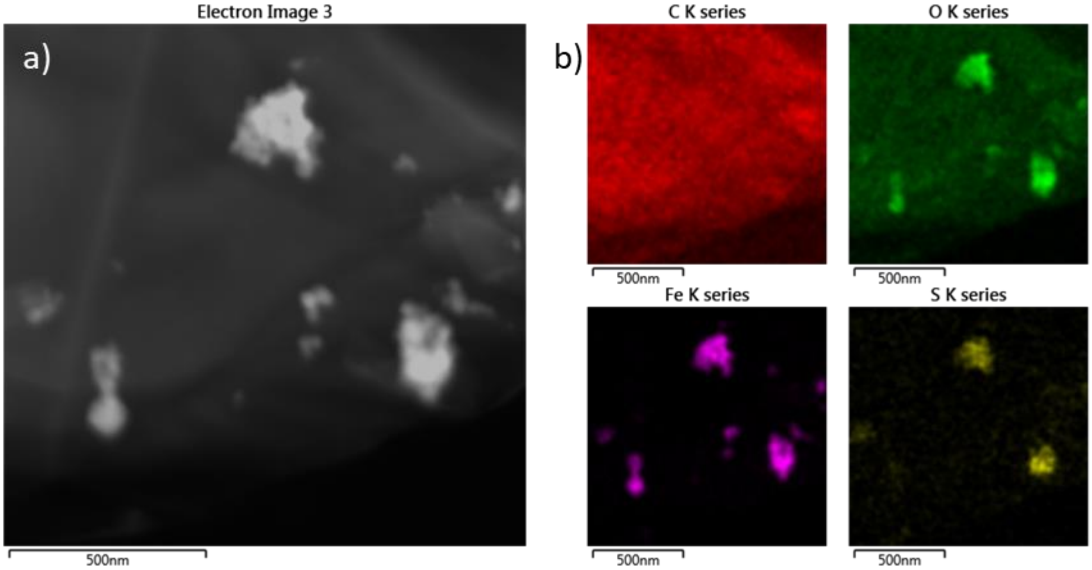

Figure S34. a) SEM images of [FeGO-a] (H_2 reduced) and b) EDX mapping analysis.

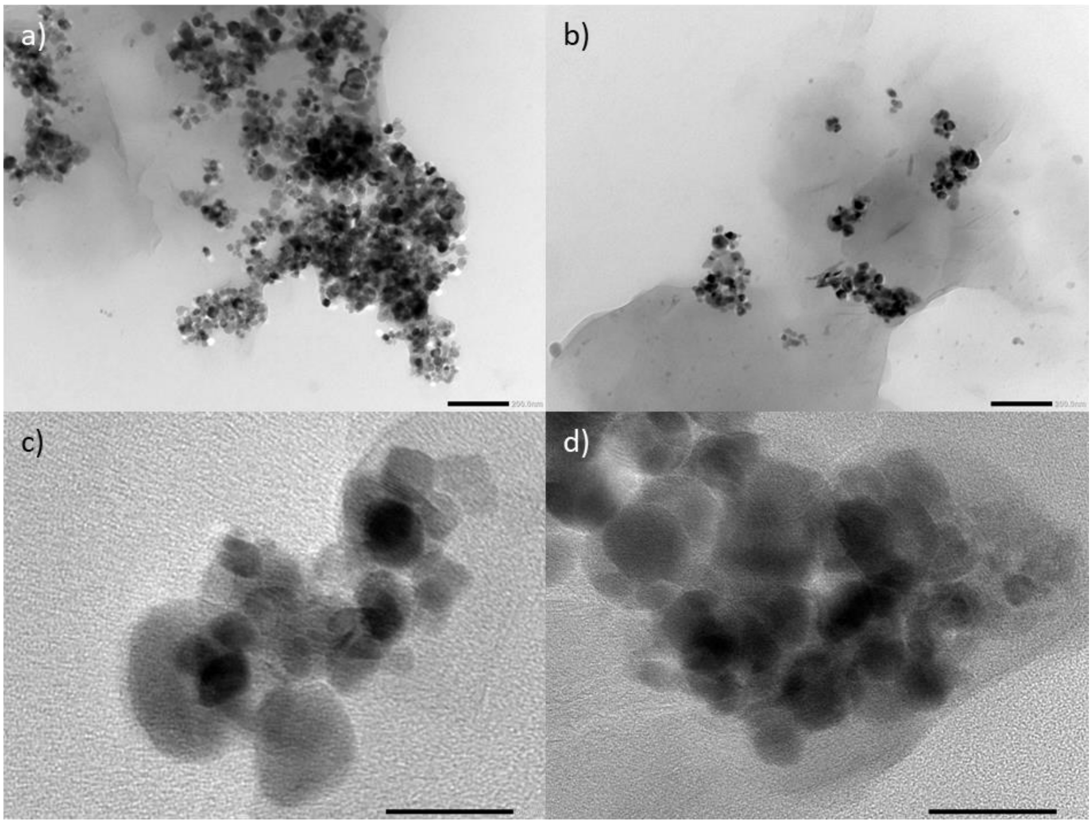

Figure S35. HRTEM images of [FeGO] (H_2 reduced) at different magnifications. a), b) Scale bar: 200 nm; c), d) scale bar: 50 nm.

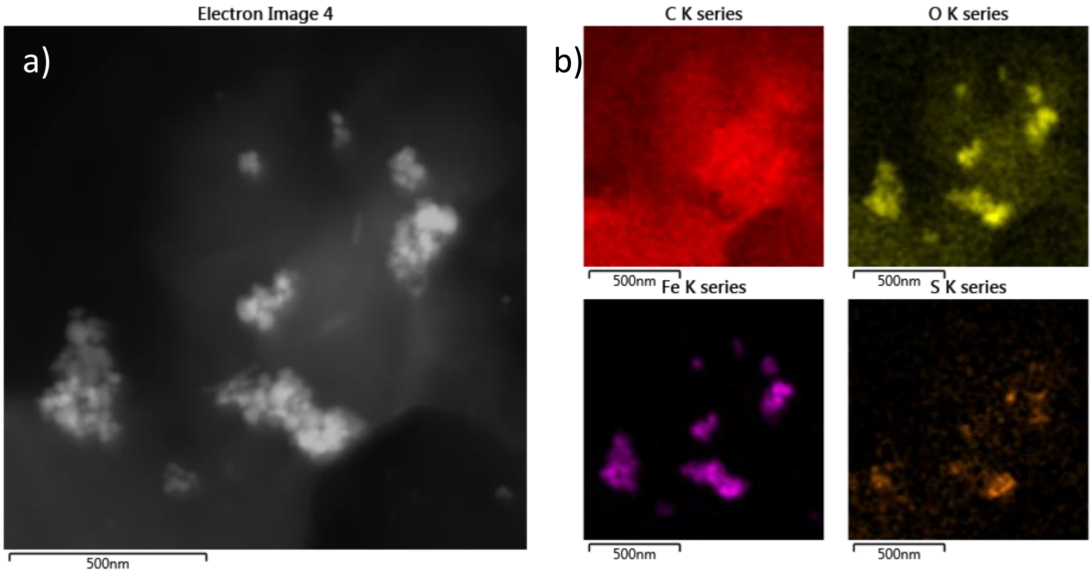

Figure S36. a) SEM images of [FeGO] (H_2 reduced) and b) EDX mapping analysis.

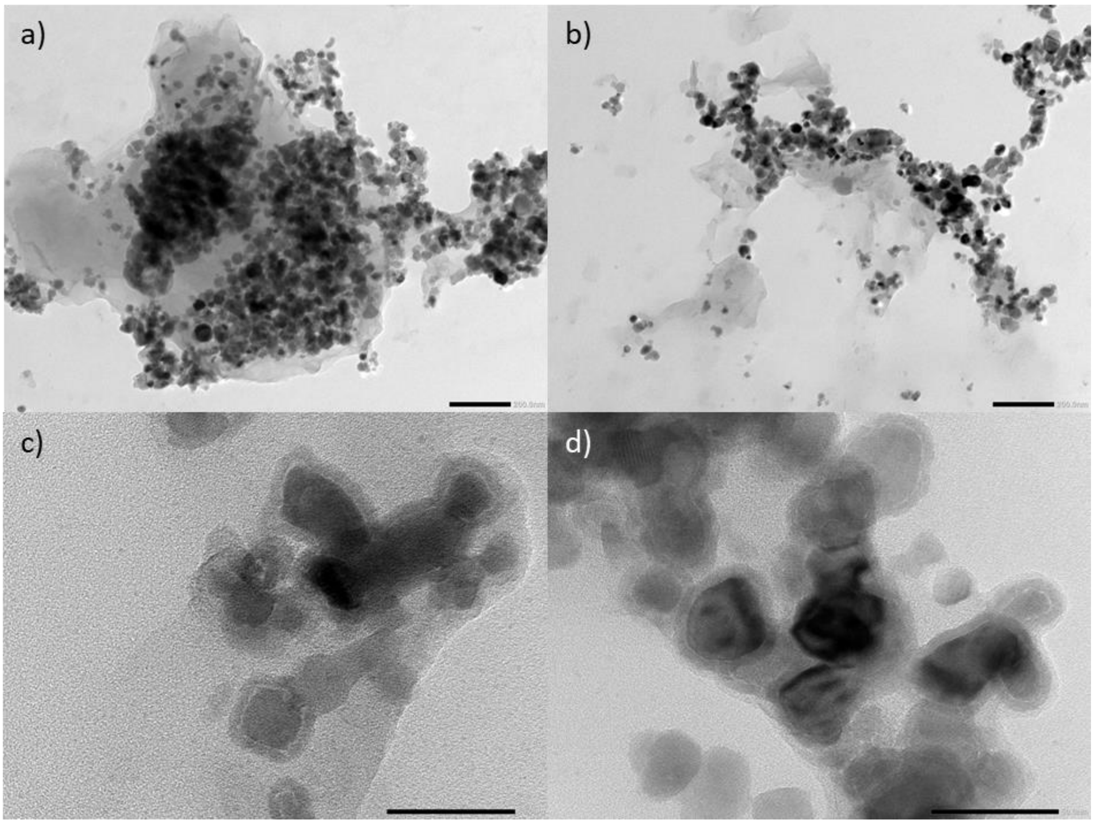

Figure S37. HRTEM images of [PdFeGO-a] (H_2 reduced) at different magnifications. a), b) Scale bar: 200 nm; c), d) scale bar: 50 nm.

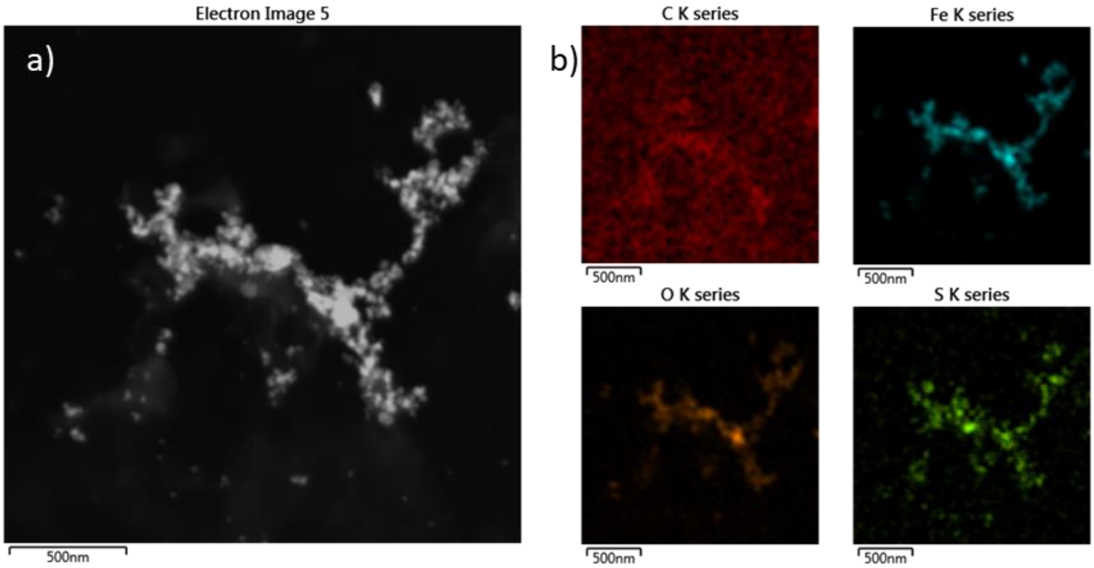

Figure S38. a) SEM images of [PdFeGO-a] (H_2 reduced) and b) EDX mapping analysis.

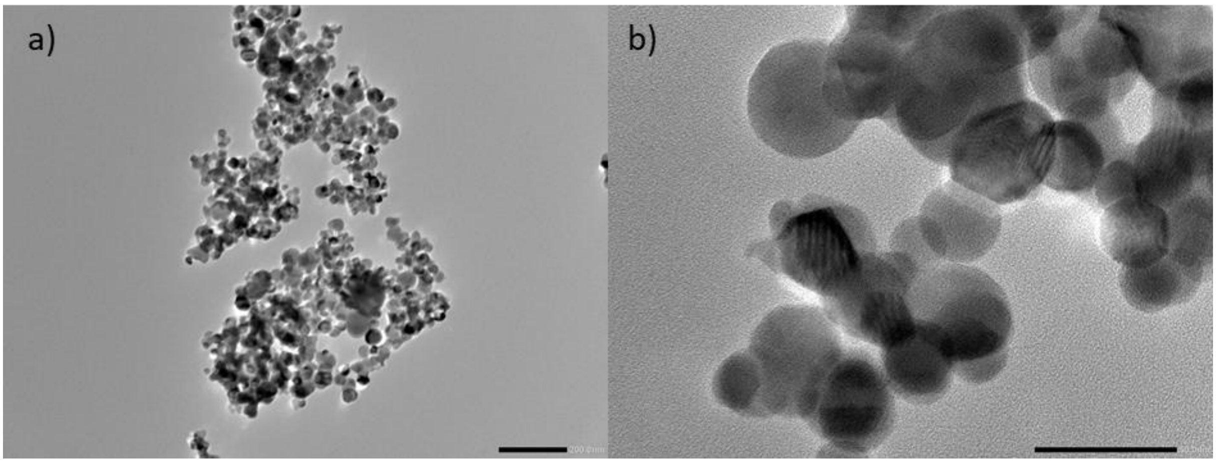

Figure S39. HRTEM images of [Fe_2O_3] particles (H_2 reduced) at different magnifications. a) Scale bar: 200 nm; b) scale bar: 50 nm.

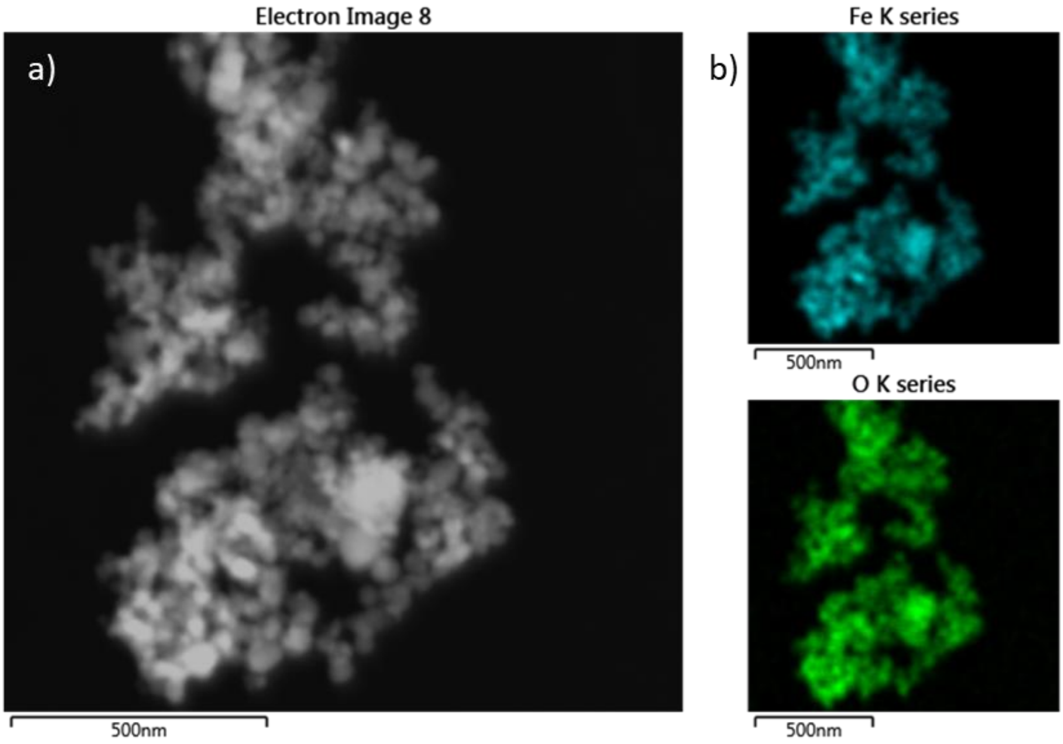

Figure S40. a) SEM images of [Fe_2O_3] particles (H_2 reduced) and b) EDX mapping analysis.

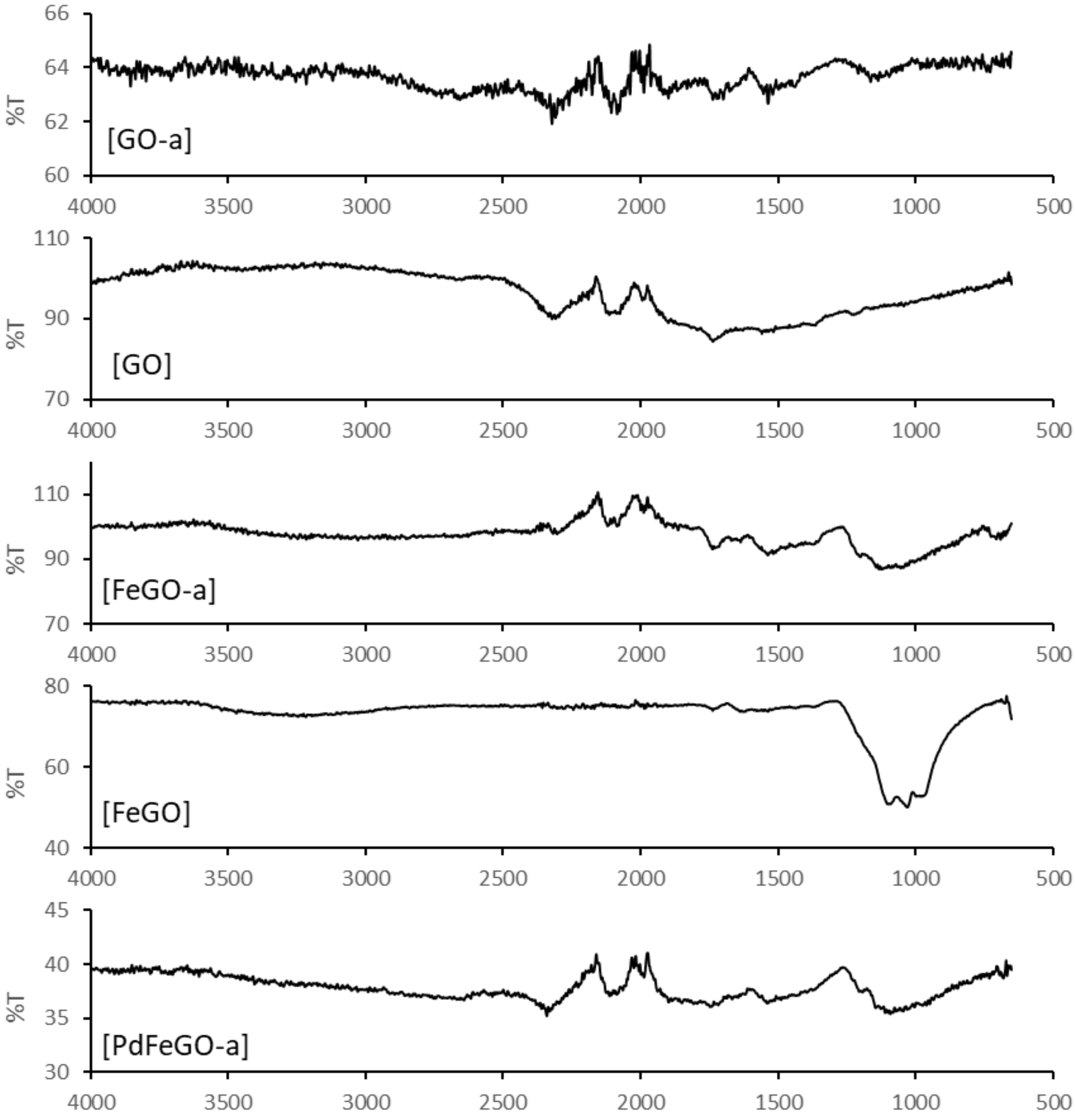

Figure 41. Spectroscopic characterisation by FTIR for the precursors and pre-catalysts after H_2 reduction.

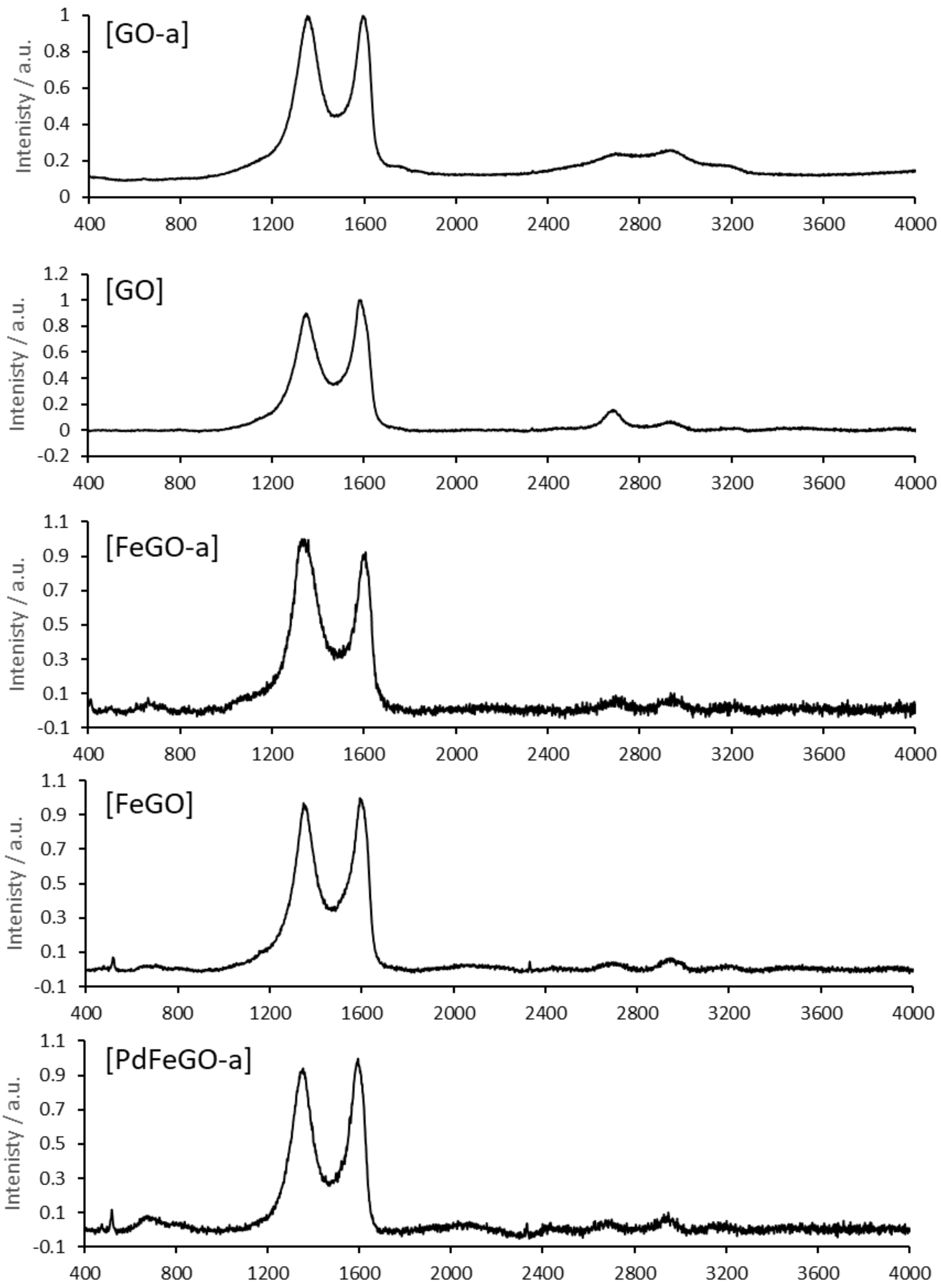

Figure 42. Raman **s**pectroscopy characterisation of the precursors and pre-catalysts after H_2 reduction.

References:

1. M. J. McAllister, J.-L. Li, D. H. Adamson, H. C. Schniepp, A. A. Abdala, J. Liu, M. Herrera-Alonso, D. L. Milius, R. Car and R. K. Prud'homme, *Chem. Mater.*, 2007, **19**, 4396-4404.
2. Z.-S. Wu, W. Ren, L. Gao, B. Liu, C. Jiang and H.-M. Cheng, *Carbon*, 2009, 47, 493-499.
3. X. Wang, L. Zhi and K. Müllen, *Nano Lett.*, 2008, 8, 323-327.
4. X. Li, H. Wang, J. T. Robinson, H. Sanchez, G. Diankov and H. Dai, *J. Am. Chem. Soc.*, 2009, 131, 15939-15944.
5. M. Lledos, V. Mirabello , S. Sarpaki, H. Ge, H. J. Smugowski, L. Carroll, E. O. Aboagye, F. I. Aigbirhio, S. W. Botchway, J. R. Dilworth, D. G. Calatayud, P. K. Plucinski, G. J. Price, S. I. Pascu, ChemNanoMat 2018,4, 361–372

5 Handbook of Graphene: Energy, Healthcare, and Environmental Applications, Wiley 2019, Edited by Cengiz Ozkan, Umit S. Ozkan; Chapter 13. Authors: D.G. Calatayud, F. Cortezon-Tamarit, B. Mao, V. Mirabello, S. I. Pascu

6. R. E. Owen, J. P. O’Byrne, D. Mattia, P. Plucinski, S. I. Pascu, and M. D. Jones ChemPlusChem, 2013,78, 1536.
7. Justin P O'Byrne, Rhodri E Owen, Daniel R Minett, Sofia I Pascu, Pawel K Plucinski, Matthew D Jones, Davide Mattia, Catalysis Science & Technology, 2013, 3(5), 1202
